# Quantitative Phenotype Morbidity Description of *SATB2*-Associated Syndrome

**DOI:** 10.1155/2023/8200176

**Published:** 2023-04-26

**Authors:** Yuri A. Zarate, Katherine Bosanko, Amrit Kannan, Ashlen Thomason, Beth Nutt, Nihit Kumar, Kirt Simmons, Aaron Hiegert, Larry Hartzell, Adam Johnson, Tabitha Prater, Eduardo Pérez-Palma, Tobias Brünger, Arthur Stefanski, Dennis Lal, Aisling R. Caffrey

**Affiliations:** ^1^Division of Genetics and Metabolism, University of Kentucky, Lexington, KY, USA; ^2^Section of Genetics and Metabolism, University of Arkansas for Medical Sciences, Little Rock, AR, USA; ^3^University of Arkansas for Medical Sciences School of Medicine, Little Rock, AR, USA; ^4^Audiology/Speech Pathology Department, Arkansas Children's Hospital, Little Rock, AR, USA; ^5^Division of Child and Adolescent Psychiatry, University of Arkansas for Medical Sciences, Little Rock, AR, USA; ^6^Department of Pediatric and Special Needs Dentistry, University of Arkansas for Medical Sciences, Little Rock, Arkansas, USA; ^7^Department of Otolaryngology, Head and Neck Surgery, University of Arkansas for Medical Sciences, Little Rock, Arkansas, USA; ^8^Department of Clinical Nutrition, Arkansas Children's Hospital, Little Rock, AR, USA; ^9^Universidad del Desarrollo, Centro de Genética y Genómica, Facultad de Medicina Clínica Alemana, Santiago, Chile; ^10^Cologne Center for Genomics, University of Cologne, Cologne, NRW, Germany; ^11^Genomic Medicine Institute, Lerner Research Institute, Cleveland Clinic, USA; ^12^Epilepsy Center, Neurological Institute, Cleveland Clinic, Cleveland, USA; ^13^Stanley Center for Psychiatric Research, Broad Institute of MIT and Harvard, Cambridge, MA, USA; ^14^Analytic and Translational Genetics Unit, Massachusetts General Hospital, Boston, MA, USA; ^15^Health Outcomes, College of Pharmacy, University of Rhode Island, Kingston, RI, USA

## Abstract

Characterized by developmental delay with severe speech delay, dental anomalies, cleft palate, skeletal abnormalities, and behavioral difficulties, *SATB2*-associated syndrome (SAS) is caused by pathogenic variants in *SATB2*. The SAS phenotype range of severity has been documented previously in large series. Using data from the SAS registry, we present the SAS severity score, a comprehensive scoring rubric that encompasses 15 different individual neurodevelopmental and systemic features. Higher (more severe) systemic and total (sum of neurodevelopmental and systemic scores) scores were seen for null variants located after amino acid 350 (the start of the CUT1 domain), the recurrent missense Arg389Cys variant (*n* = 10), intragenic deletions, and larger chromosomal deletions. The Arg389Cys variant had the highest cognitive, verbal, and sialorrhea severity scores, while large chromosomal deletions had the highest expressive, ambulation, palate, feeding and growth, neurodevelopmental, and total scores. Missense variants not located in the CUT1 or CUT2 domain scored lower in several subcategories. We conclude that the SAS severity score allows quantitative phenotype morbidity description that can be used in routine clinical counseling. Further refinement and validation of the SAS severity score are expected over time. All data from this project can be interactively explored in a new portal.

## 1. Introduction

*SATB2*-associated syndrome (SAS; Glass syndrome, OMIM 612313) is a multisystemic autosomal dominant disorder caused by a variety of different molecular alterations involving *SATB2* [1]. While predominantly a neurodevelopmental disorder, other systemic features distinguish SAS from overlapping conditions. Consistently described features include developmental delay with severe speech compromise, behavioral abnormalities, facial dysmorphism, palatal anomalies, dental problems, decreased bone density, hypotonia, growth retardation, and epilepsy [1–3].

With an estimated incidence of approximately 1 in 30,000 births [4], it has become increasingly clear over the last several years that SAS is one of the most common causes of unexplained developmental disorders [5, 6]. With the identification of hundreds of individuals with SAS, the range of phenotypic variability continues to expand. Although speech delay is one of the main features of SAS, studies have shown that despite the large proportion of primarily nonverbal communicators, a few retain some verbal ability to communicate [7]. Likewise, composite nonverbal cognitive scores can display variability from mild to severe [3, 7]. The broad range in severity can also be found for many of the other systemic features, including seizures (some individuals have severe epileptic encephalopathy), behavioral problems (antipsychotic medications or management at inpatient facilities for mental health issues have been reported), sleeping difficulties (including the need for high doses of multiple medications to aid with sleep), skeletal complications (some individuals have had recurrent fractures requiring treatment with medications for metabolic bone issues), dental anomalies (significant primary and secondary dentition problems have been reported), and growth delay (some individuals have needed aggressive feeding interventions) [8–17].

Genotype-phenotype correlations in SAS are still broad and limited to mutation categories. Individuals with large deletions are more likely to have growth retardation. In contrast, those with missense pathogenic variants have a lower frequency of cleft palate, but a higher incidence of seizures, and those with frameshift variants are more likely to have feeding difficulties [1]. The lack of an objective tool to assess disease severity has thus far made genotype-phenotype correlation difficult. It will be a major hindrance to assessing the outcomes of interventions in potential future clinical trials. Recognizing the heterogeneity seen in individuals with SAS from our experience at the *SATB2*-associated syndrome international clinic at Arkansas Children's Hospital, a referral center for individuals with SAS, we propose a severity-scoring system for the quantitative and standardized assessment of clinical severity in individuals with SAS.

## 2. Materials and Methods

### 2.1. Participants

A phenotypically and genetically heterogeneous cohort of 164 individuals with a molecularly confirmed diagnosis of SAS were enrolled under a research clinical registry protocol approved by the Institutional Review Board of the University of Arkansas for Medical Sciences (Protocol #205083). Written informed consent was obtained from all participants. For all individuals, medical records, including laboratory results, were reviewed with supplementary information also obtained by a parental report through a REDCap™ questionnaire. Many of these individuals have also been followed in a dedicated SAS clinic hosted at Arkansas Children's Hospital, where they undergo a comprehensive multidisciplinary clinical evaluation, often with other laboratory and imaging studies performed.

### 2.2. Severity Score

A severity score was developed to quantitatively analyze the severity of the phenotype in individuals with SAS (Table S1). The SAS severity score is an organ system-based rubric that includes 15 different individual ordinal features (“categories”) measuring common phenotypic traits in SAS and grouped into neurodevelopmental and systemic. In turn, neurodevelopmental, systemic, and total (sum of neurodevelopmental and systemic) scores were derived. Clinical findings for the score were chosen based on their medical relevance according to our experience through the SAS clinic and considering previous parental feedback obtained by the SATB2 Gene Foundation (http://www.satb2gene.org) aimed at determining the most impactful difficulties faced in SAS.

Scores range from 0 to 2 for some features to as high as 0 to 5 for the most impactful affected systems, with 0 representing the least severe (conversely, higher severity scores indicate increasing phenotypic severity). All study participants were scored based on objective assessments obtained through the SAS multidisciplinary clinic, whenever available, or the medical information retrieved from patient records and parental interviews as an alternative. IQ scores were used preferentially for the cognitive/adaptive skill domain, with other adaptive and developmental scores used only if unavailable. Due to the nature of the neurodevelopmental questions, the SAS severity score was only calculated for individuals three years of age and older.

### 2.3. Molecular Data

We collected the type of molecular alteration, location within the gene, and predicted consequences on the gene product. All participants in this study had molecular confirmation of their diagnosis and were classified into three broad categories: null variants, missense variants, and chromosomal anomalies. Null pathogenic variants were defined as those predicted to result in an absent or functionally abnormal gene product, including nonsense (encoding a premature termination codon), frameshift (altering the reading frame), or splice-site (canonical ±2 splice sites) variants. Exonic deletions (single or multiexonic) were also classified as null variants for data analysis purposes. Missense variants, defined as those encoding an alternative but functional protein product, were classified according to the American College of Medical Genetics and Genomics (ACMG) variant-interpretation guidelines [18]. Lastly, large contiguous gene deletions and duplications were classified as chromosomal. One individual (SATB2 ID#178) with an intronic variant was included in the overall analysis.

### 2.4. Data Analysis

Basic descriptive statistics were performed, with means standard deviations, confidence intervals estimated for continuous traits and scores, and frequencies for discrete traits. Continuous traits were examined for normality. Where items contributing to the severity score were unavailable, a default score of “0” was given in the corresponding category before an overall severity score was calculated. Differences in mean severity scores between males and females, age groups (3–5 years, 6–10 years, 11–17 years, and ≥18 years), and variant categories (null, missense, and chromosomal) were compared statistically with analysis of variance (ANOVA).

To compare scores (scores for each individual category of the scoring rubric, neurodevelopmental severity scores, systemic severity scores, and total severity scores) between variant categories, adjusted means and 95% confidence intervals, controlling for sex and age group, were calculated with multiple linear regression. Separate models were run for each score and variant comparison, and variant categories with less severe scores were selected as the reference group. The variant category was compared as null, missense (reference), and chromosomal. We further subdivided the molecular categories into 11 subcategories for comparison: (1) missense variant p.Arg389Cys, (2) missense variants (different than p.Arg389Cys) located in the CUT1 domain, (3) missense variants located in the CUT2 domain, (4) missense variants located in the HOX domain (reference), (5) other missense variants (not located in the CUT1, CUT2, or HOX domains), (6) null variants before amino acid 350 (the start of the CUT1 domain), (7) null variants at or after amino acid 350, (8) chromosomal deletions of less than 6 Mb, (9) chromosomal deletions of 6 or more Mb in size, (10) intragenic deletions, and (11) splice variants.

As the severity-scoring scale of each category of the rubric varied (some categories range from 0 to 2 points, others from 0 to 5 points), for visual comparison of the scores via radar charts, we calculated standardized adjusted means by first standardizing each score and then calculating sex- and age-group-adjusted means of the standardized scores. Statistical significance was set at *p* value < 0.05, except for comparisons of the adjusted means of the individual domains (*n* = 15), where statistical significance was set at *p* < 0.0033 per Bonferroni adjustment. We utilized SAS (Version 9.4, Cary, NC) and GraphPad Prism (v9.3.1.) for all statistical analyses.

### 2.5. Portal Design

For the SATB2 Portal, we used the Shiny R (1.7.1) framework from RStudio (https://shiny.rstudio.com/) to build the interactive web portal for compatibility, expendability, and portability. The portal is publicly available, hosted at the Broad Institute, and was deployed with Google Cloud service using a self-contained Docker image. The code is available on GitHub (https://github.com/LalResearchGroup/SATB2_Portal).

## 3. Results

### 3.1. Cohort Description

As of April 2022, 164 individuals from 19 different countries were enrolled in the international *SATB2*-associated syndrome registry for whom a SAS score could be calculated (supplementary file). Of these, 86 (52.4%) were male, and the median age at collection was 8.5 years (3–38 years) (Table S2). Single-nucleotide variants were found in 122 individuals (74.4%, 20 previously unreported variants in 23 individuals) (Figure S1), with the remaining having intragenic deletions or larger chromosomal abnormalities (13 previously unpublished). Novel coding variants were submitted to ClinVar (SCV002817103–SCV002817112). Forty-eight individuals harbored one of 14 recurrent variants (seen in 2 or more individuals), and the most common variant was the missense variant p.Arg389Cys (*n* = 10) located in the CUT1 domain. Null pathogenic variants were seen in 93 individuals (56.7%), while 45 individuals (27.4%) had 23 different missense alterations. Most missense variants found in the cohort (39/45 = 87%) were de novo. All 23 different missense variants were classified as either likely pathogenic or pathogenic based on ACMG guidelines except for Leu545Pro, which remained classified as of unknown significance. However, this individual (SATB2-212) was included in the analysis as she displayed neurodevelopmental and dental phenotypes consistent with SAS.

With a total possible maximum score of 47, the mean score was 19.6 (SD = 6.2, range 2–34). Neurodevelopmental, systemic, and total severity scores tended to be highest in individuals older than ten years of age (*p* = 0.07, 0.25, and 0.07, respectively) with no differences by sex. Total severity scores were slightly higher for individuals with null variants, but this was not statistically significant (*p* = 0.44) (Table 1).

### 3.2. Severity Scores for Recurrent Variants

Severity scores were analyzed for variants present in 3 or more individuals. Variants Arg429Gln (*n* = 5) and Ser649Leu (*n* = 4) had the highest and lowest adjusted mean scores, respectively: (1) neurodevelopmental 14.80; 95%CI = 10.97, 18.62, and 9.47; 95%CI = 5.17 and 13.77; (2) systemic 8.55, 95%CI = 6.07, 11.03, and 3.56; 95%CI = 0.77 and 6.36; and (3) total 23.35,95%CI = 18.00, 28.69, and 13.03; 95%CI = 7.02 and 19.04. Systemic and total severity scores were statistically significantly higher for Arg429Gln (*n* = 5), Arg389Cys (*n* = 10), and all other mutations (*n* = 130) compared with the reference, Ser649Leu (*n* = 4).

### 3.3. Severity Score by Genotype

**Figure 1:**
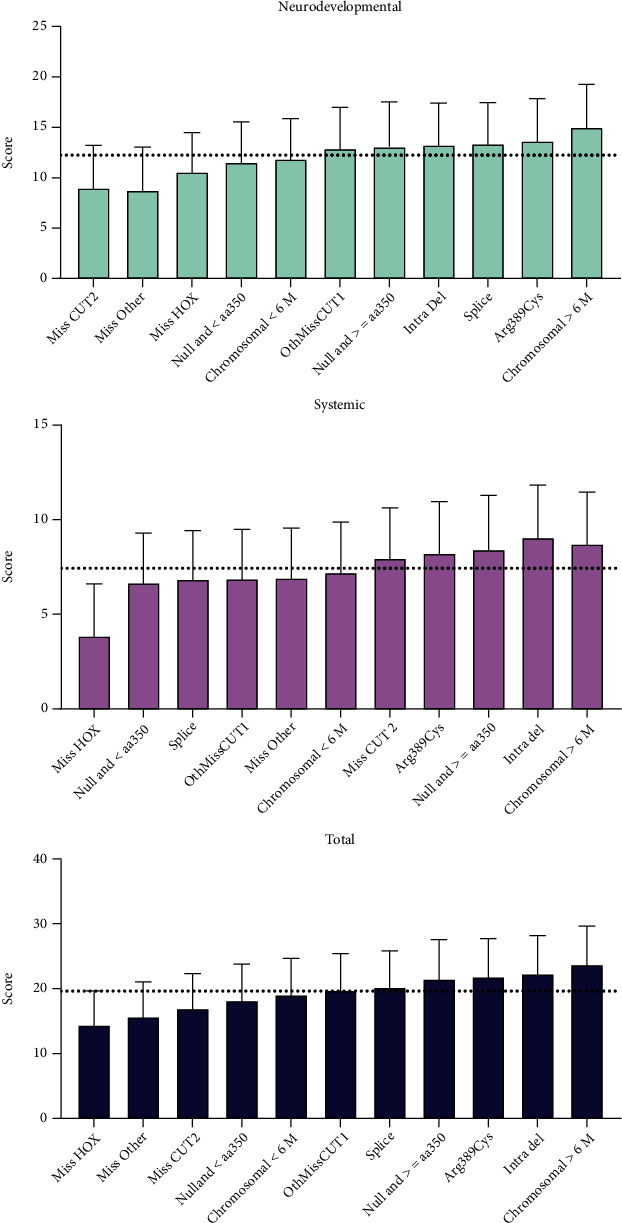
Average adjusted neurodevelopmental (top), systemic (middle), and total (bottom) severity scores by molecular subcategory. Higher severity scores (groups to the right) indicate higher phenotypic severity. The dashed line represents the average across all individuals.

**Figure 2:**
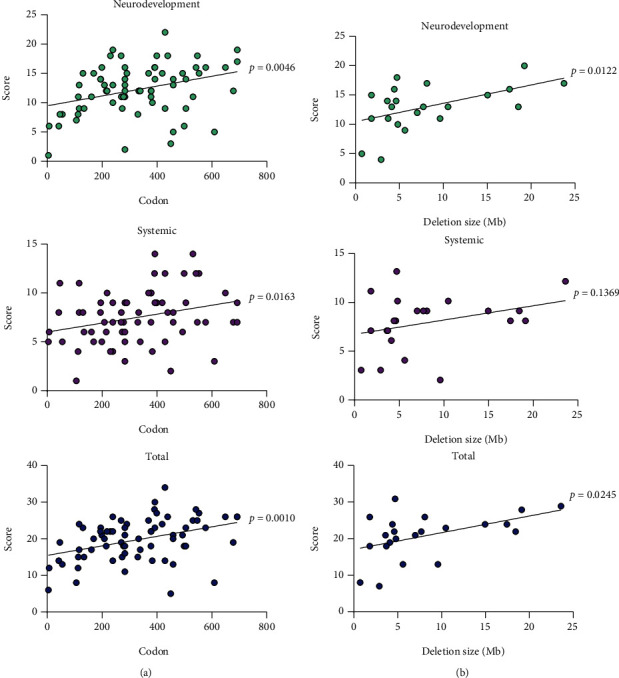
Adjusted neurodevelopmental, systemic, and total severity scores for individuals with predicted null coding variants (a) and chromosomal deletions (b). Severity scores increase by codon location downstream (*n* = 69) and by the size of the chromosomal deletion (*n* = 22).

In-depth analysis according to the mutation subgroup revealed statistically significant higher systemic and total scores for null variants located after amino acid (aa) 350 (the start of the CUT1 domain), the common missense Arg389Cys variant, intragenic deletions, and larger-than-6 Mb chromosomal deletions when compared to the lowest scoring group (missense variants located in the HOX domain) (Figure 1 and Tables S3–S5). For null variants, there was a tendency to have higher neurodevelopmental (slope *p* = 0.0046), systemic (slope *p* = 0.0163), and total (slope *p* = 0.001) scores the deeper the change went into the coding region (Figure 2(a)). Likewise, there was a correlation with the deletion size, with individuals having higher neurodevelopmental (slope *p* = 0.0122) and total (slope *p* = 0.0245) scores as deletion sizes increased (Figure 2(b)).

### 3.4. Individual Clinical Category Severity by Genotype

**Figure 3:**
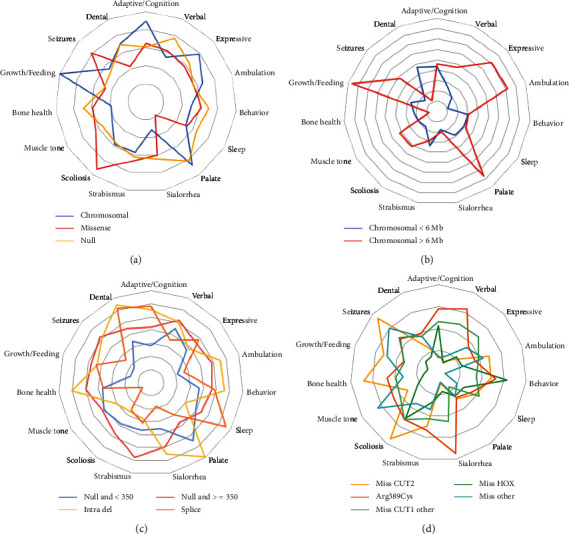
Radar charts showing the 15 clinical categories used in the *SATB2*-associated syndrome severity score. (a) Scores by broad categories. (b) Scores for individuals with chromosomal anomalies by size. (c) Scores for individuals with predicted null variants. (d) Scores for individuals with missense variants.

The results for each molecular group for the combined scores, and all the categories in the scoring rubric are summarized in Tables S6 to S20. The standardized mean scores for all categories are plotted on a “radar” chart in Figure 3, highlighting phenotype variability between molecular groups.

Individuals with chromosomal abnormalities had significantly higher palate and feeding and growth scores while individuals with missense variants had higher scoliosis scores. The missense variant p.Arg389Cys had the highest (most severe) cognitive, verbal, and sialorrhea severity score. In contrast, chromosomal deletions larger than 6 Mb had the highest expressive, ambulation, palate, feeding and growth, neurodevelopmental, and total scores. Missense variants in the HOX domain scored the lowest (least severe) in the verbal, ambulation, strabismus, bone, feeding and growth, seizure, and dental categories, with the lowest systemic and total scores. Missense variants outside the main domains (CUT1, CUT2, and HOX) scored lowest in the cognition, behavior, sleep, and sialorrhea categories, with the lowest neurodevelopmental score.

### 3.5. Public Access to Severity Scores: the SATB2 Portal

To enable others to use our rich data for research and education, we developed the SATB2 Portal (https://satb2-portal.broadinstitute.org/) (Figure S2). All aggregated data is shared according to the FAIR principles to make it findable, accessible, interoperable, and reusable [19]. The SATB2 Portal is an interactive and user-friendly web application that combines genetic and clinical data of individuals with *SATB2*-associated syndrome with experimental functional and annotation data on variants. Users can navigate through four sections: (1) Basic Information, (2) Families, (3) Variant Analysis and Severity Scores, and (4) Genotype-Phenotype. Integrating and connecting these data through the portal infrastructure enables users to explore genotype to structure, phenotype, and severity score associations (Figures S3 and S4).

## 4. Discussion

Considering the broad range of phenotypic variation known to be present in SAS, we present an objective tool aimed at quantifying severity using a scoring rubric that encompasses the clinical domains most frequently affected and that have the greatest impact on the quality of life in this population. The scoring system can be applied using either routine and accessible clinical data or objective patient information when available. To make all aggregated data available for educational purposes and research projects, we developed the SATB2 Portal (https://satb2-portal.broadinstitute.org/). In addition to data access, we provide a novel severity score framework that facilitates the evaluation of genetic and clinical features of the *SATB2*-associated syndrome.

The greatest strength of this study is the use of a large dataset derived from the international SAS registry to provide the basis for clinical counseling. Although the study lacked the power to identify statistically significant differences among individuals with specific recurrent pathogenic variants, we present the extremes of the severity spectrum. As a group, while there were no sex differences, severity scores were, in general, higher in older individuals. With age, individuals with SAS may improve in their ambulation and communication categories (and score lower) but are also more likely to have the compromise of other systems evaluated in the rubric that become only apparent (or actively screened) in older ages.

Analysis by broad mutation categories (chromosomal, missense, or null) did not reveal significant differences in the total scores. However, the variation in the scores for palate, scoliosis, and growth/feeding was noted. Further review by mutation subgroups revealed higher scores for individuals with null variants located after amino acid 350, the common missense Arg389Cys variant, intragenic deletions, and chromosomal deletions larger than 6 Mb. Following previously reported work, the phenotype subcategories [20] showed that individuals with chromosomal abnormalities had higher feeding and growth scores. We found a direct relationship between deletion size and severity that is likely related to other contiguous genes included in the deleted segment. However, the reason for higher severity in the other molecular subcategories is less apparent. Previous studies have documented that the CUT1 domain is essential for binding to the matrix-associated region (MAR) and that the Arg389Cys variant, in particular, has a more diffuse nuclear localization pattern compared to other missense variants [2]. In our study, individuals with missense variants in the HOX domain and those not located in one of the main domains (CUT1, CUT2, and HOX) had lower scores. This correlates with the variability in severity seen in individuals with the closely related syndrome caused by *SATB1* dysfunction in which missense variants in the CUT1 and CUT2 DNA-binding domains often have a more severe phenotype as well [21]. Accordingly, we theorize that the *SATB2* Arg389Cys variant has an increased transcriptional repressive effect to explain the perceived more severe phenotype. For null variants, we found a correlation between protein length and severity (higher scores for more distal variants). Previous studies have documented that other proximal variants can still be translated besides the stop-gain variants located in the in the last exon [2, 22]. How these shorter truncated proteins lead to variable phenotypes remains unclear. Other genotype-phenotype correlations were also noted when analyzing each phenotype category of the scoring rubric. Often, variants near each other (and sometimes with the same variant) can result in substantially different severity. Lastly, the recurrent Ser649Leu variant located in the HOX domain appears to have a less severe impact as demonstrated by the lower neurodevelopmental and systemic score, but this observation will need to be replicated with a larger cohort. We speculate that variants located in the HOX domain could have a different type of functional effect compared to those affecting the CUT1 and CUT2 domains to explain the phenotypic differences. Taken together, we postulate that there are different mutation-specific mechanisms (including both loss and gain of function) which determine the degree of severity in SAS.

Major limitations of this study include the relatively small number of individuals, particularly for the less common pathogenic variants, and the potential inaccuracies from the parent-reported information. Although complete medical records and/or in-person evaluations by our team were available for most individuals, detailed medical information to verify each subcategory of the scoring rubric was not always available. In very few instances, some parameters of the scoring rubric could not be completed, so a score of zero was assigned. This could lead to lower scores for individuals in whom less information was available. While the international registry includes participants from all continents and the invitation to participate in the severity score was distributed among them, the severity score was calculated in a subset of individuals that would have at least acceptable English- or Spanish-reading skills and with access to a computer with an internet connection to complete the accompanying survey questions. Despite this limitation, given the genetically heterogeneous population included, we do believe our data could be carefully extrapolated to other populations less represented in our data. Lastly, it is important to acknowledge that there are few validated severity scales for rare genetic neurodevelopmental disorders. We developed the SAS severity score through consensus from a large multidisciplinary team with vast expertise in caring for individuals with SAS and from caregiver surveys.

The SAS severity score represents a step forward in the quantitative phenotypic morbidity description seen in SAS which can be used in routine clinical counseling. The SATB2 Portal will facilitate access to the scoring rubric for providers and families. We acknowledge that the parent report and the objective components of the SAS severity score still need to be refined and validated over time as more individuals with SAS are diagnosed. This will be particularly important when considering its role in the design and outcome measures in future clinical trials. Concurrent advances in understanding the functional consequences of the different molecular alterations responsible for SAS will be needed to integrate the genotype and phenotype fully.

## Figures and Tables

**Table 1:** Demographics and comparison of the *SATB2*-associated syndrome severity score overall and by age group and sex.

	*n*	Neurodevelopment		Systemic		Total	
		Mean (SD)	*p* value	Mean (SD)	*p* value	Mean (SD)	*p* value
Total	164	12.19 (4.38)		7.44 (2.87)		19.63 (6.17)	
Age							
3 to 5 years	29	12.24 (3.73)	0.07	6.86 (2.89)	0.25	19.10 (5.13)	0.07
6 to 10 years	70	11.21 (4.97)		7.21 (3.10)		18.43 (7.05)	
11 to 17 years	41	13.20 (3.96)		8.12 (2.68)		21.32 (5.59)	
≥18 years	24	13.25 (3.43)		7.66 (2.35)		20.92 (4.80)	
Sex							
Male	86	12.60 (4.31)	0.14	7.16 (2.75)	0.37	19.77 (6.12)	0.67
Female	78	11.73 (4.44)		7.75 (2.99)		19.49 (6.27)	
Molecular^†^							
Null	93	12.45 (4.28)	0.72	7.71 (2.76)	0.33	20.16 (5.81)	0.44
Missense	45	11.82 (4.48)		6.93 (2.98)		18.76 (6.30)	
Chromosomal	25	12.32 (4.18)		7.56 (3.00)		19.88 (6.63)	

Abbreviation: SD: standard deviation. ^†^One individual with an intronic variant not included.

## Data Availability

The data that support the findings of this study are openly available at https://satb2-portal.broadinstitute.org/ and in the supplementary material of this article. The code is available on GitHub (https://github.com/LalResearchGroup/SATB2_Portal).

## Supplementary Materials

Supplementary Materials Figure S1: schematic representation of coding variants included in this study. Figures S2–S4: screenshots of the SATB2 portal and its different input modalities. Table S1: SATB2-associated syndrome severity score. Tables S2–S20: demographics and multiple linear regression models by mutation category for individual subcategories of the score. Supplementary File: individual score and molecular and demographic data for all individuals included in the study.
