# Supplementary material for: Quantitative Phenotype Morbidity Description of SATB2-Associated Syndrome
Source: Hum Mutat. 2023 Apr 26;2023:8200176. doi: 10.1155/2023/8200176 (PMC11918880; doi:10.1155/2023/8200176)
Supplement: Supplementary Materials — Figure S1: schematic representation of coding variants included in this study. Figures S2–S4: screenshots of the SATB2 portal and its different input modalities. Table S1: SATB2-associated syndrome severity score. Tables S2–S20: demographics and multiple linear regression models by mutation category for individual subcategories of the score. Supplementary File: individual score and molecular and demographic data for all individuals included in the study. [file 8200176.f1.zip › Supplementary File Severity Paper.docx]

**SUPPLEMENTARY MATERIAL**

**Quantitative phenotype morbidity description of *SATB2*-Associated Syndrome**

Yuri A. Zarate, Katie Bosanko, Amrit Kannan, Ashlen Thomason, Beth Nutt, Nihit Kumar, Kirt Simmons, Aaron Hiegert, Larry Hartzell, Adam Johnson, Tabitha Prater, Jillian Kimberlain, Eduardo Pérez-Palma, Tobias Bruenger, Arthur Stefanski, Dennis Lal, Aisling R. Caffrey.

**Figure S1.** Schematic representation of 88 coding variants reported in 122 individuals with *SATB2*-Associated syndrome (SAS). The variant location is based on NM_015265. The number associated with the variant represents the number of times the variant was reported. Dashed vertical lines and numbers within the protein diagram represent exon boundaries (exon 3 is the first coding exon) and aminoacid position, respectively. The main domains are represented in different colors within the protein schematic. Images were constructed using ProteinPaint.

**
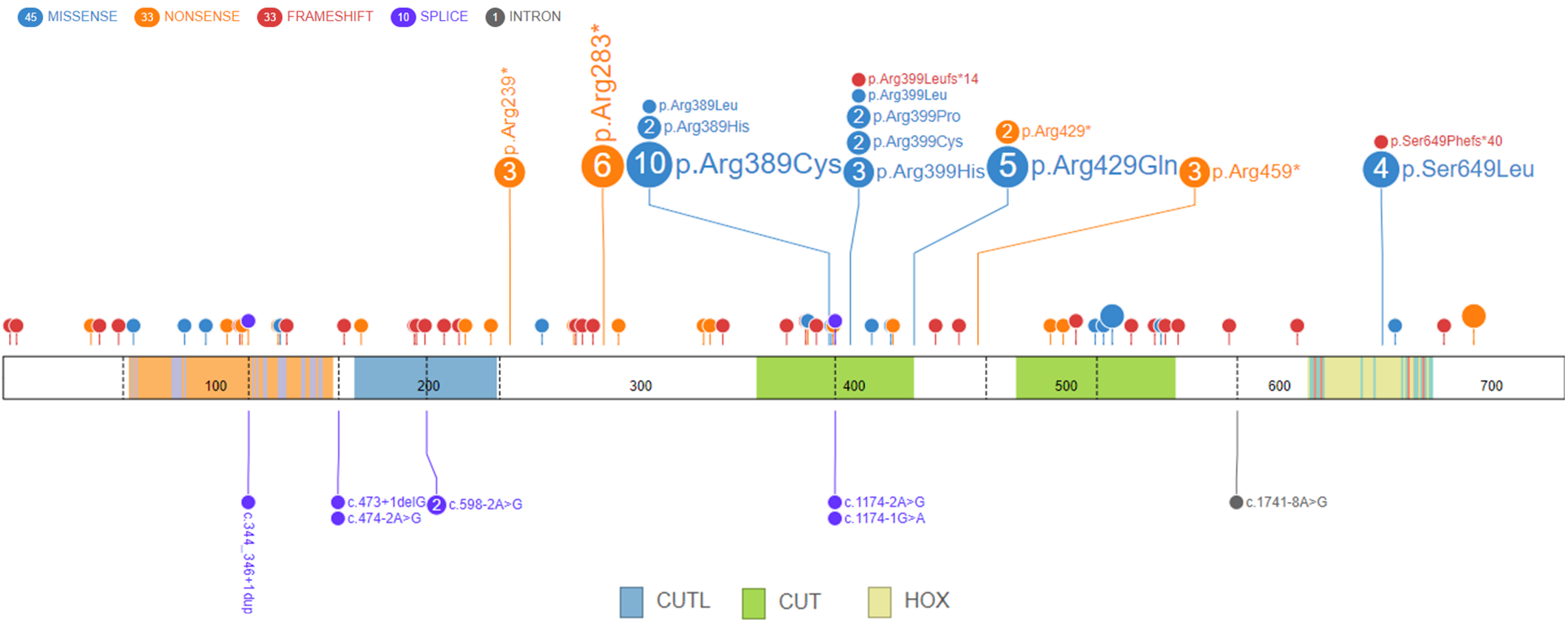
**

**Figure S2. Top.** Landing page for the SATB2 Portal. The user can explore basic information, find resources, and query the database by genotype and/or phenotype**. Bottom.** The variant analysis tab recognizes the variant by c.DNA or predicted protein alteration to offer an in-depth comparison to other individuals.

**
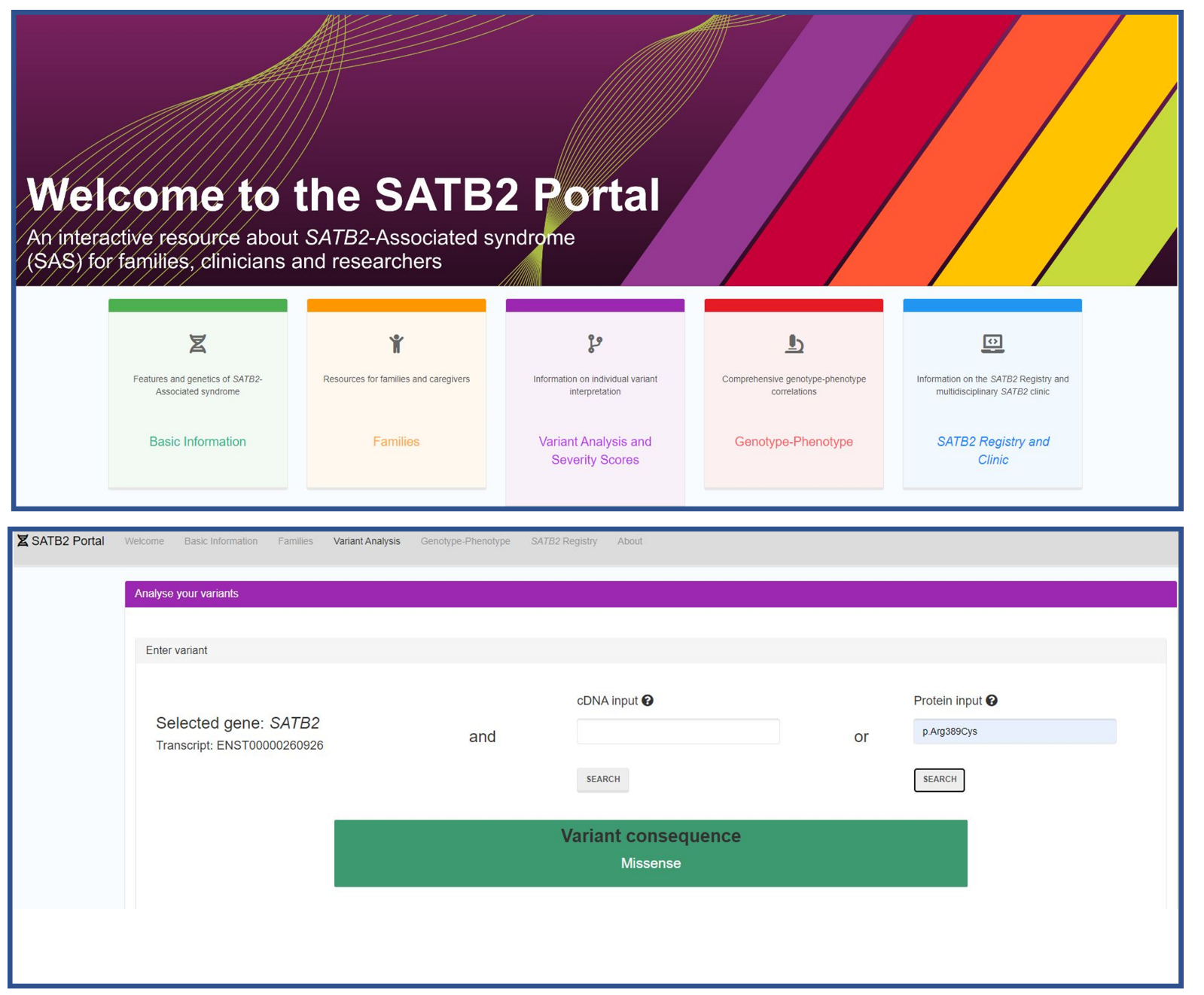
**

**Figure S3. Top.** Simple drop-down menus allow the user to score each of the 15 subcategories of the neurodevelopmental and systemic domains. An explanation of how to calculate each category is also included. **Bottom.** Aggregate scores are updated in real-time as the user enters the individual scores and comparative figures are generated allowing a quick representation of the scores for the variant of interest and how it compares to other variants of the same type, at the same protein codon, the same domain, and the overall population.

**
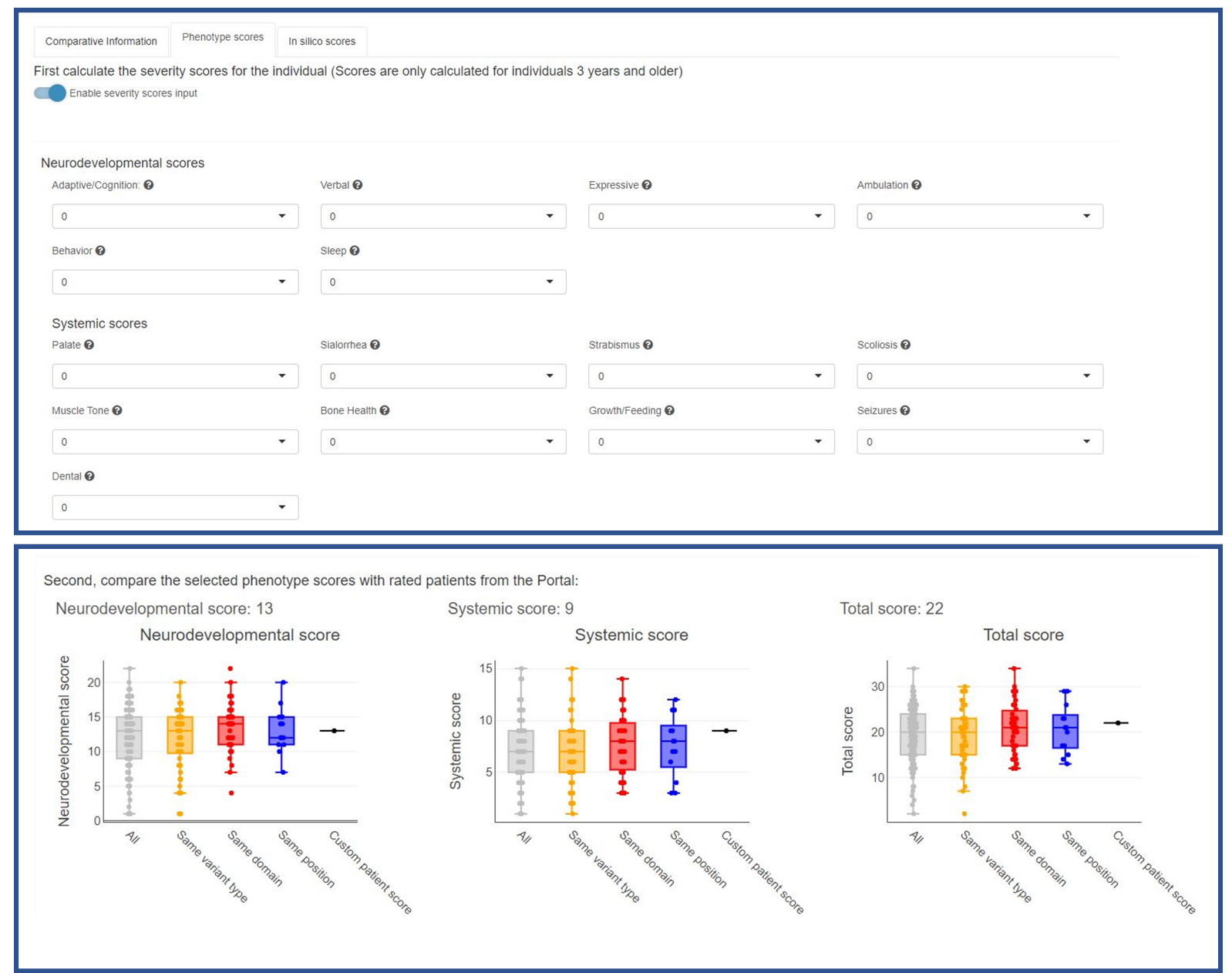
**

**Figure S4.** The portal allows for in-depth genotype-phenotype correlations. **Top.** The user can filter by variant type, specific amino acid change, or domain to obtain the desired output. **Bottom.** The frequency of phenotypic features or scores can be displayed and filtered by the genotype of interest.

**
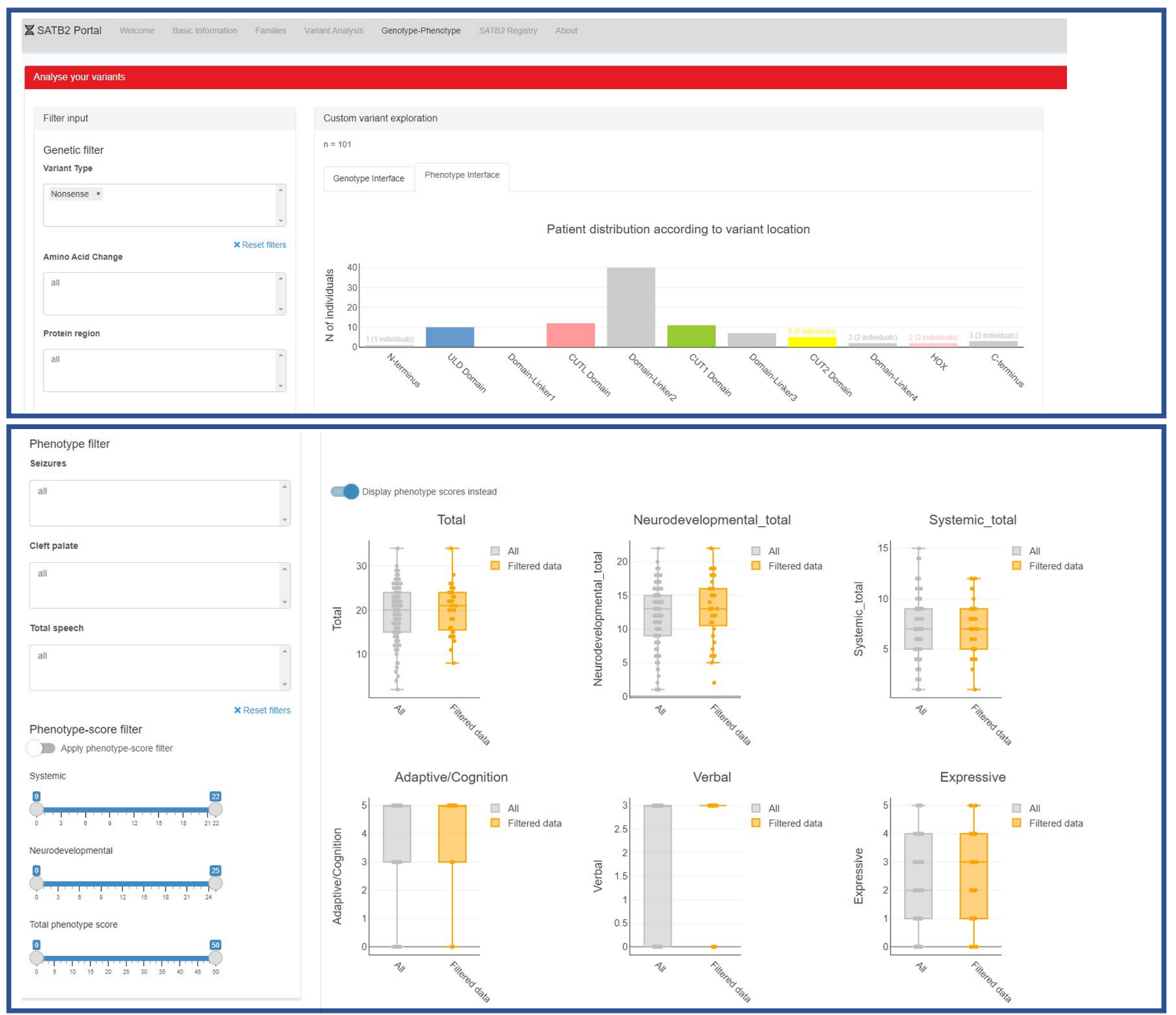
**

**Table S1.** *SATB2*-Associated syndrome (SAS) severity score.

| **Manifestation** | **Score** | **Definition** | **Alternative clinical measures and notes** |
| --- | --- | --- | --- |
| **Neurodevelopmental** |  |  |  |
| Adaptive/Cognitive | 0 | Normal cognition |  |
|  | 3 | Mild delay | IQ 51-70, or alternative^a^ |
|  | 5 | Moderate to profound delay | IQ<51 or alternative^b^ |
| Verbal language | 0 | Speaks more than 10 words |  |
|  | 3 | 10 or fewer spoken words |  |
| Expressive language | 0 | Full sentences, can participate in conversational exchanges | MLU>3 |
|  | 1 | >50 words/signs/pictures/symbols | Emergence of level VII on the Communication Matrix |
|  | 2 | 10-50 words/signs/pictures/symbols | Primarily Levels V and VI on the Communication Matrix |
|  | 3 | 1-10 spontaneous words/signs/pictures/symbols | Primarily Levels V and VI on the Communication Matrix |
|  | 4 | Communicates with some conventional gestures | Primarily Levels II through IV on the Communication Matrix |
|  | 5 | No conventional gestures | Primarily Level I or Level II on the Communication Matrix |
| Ambulation | 0 | Acquired <18 mos |  |
|  | 1 | Walks alone 18 to 29 months |  |
|  | 2 | Walks alone >29 months to 59 months |  |
|  | 3 | Walks alone >59 months (5 years) to 8 years |  |
|  | 4 | Walks alone > 8 years or older than 8 years and not acquired |  |
| Behavior | 0 | No behavioral concerns | SDQ Parent Total Score <14 |
|  | 1 | Mild (hyperactivity, poor boundaries, sensory, scratching/pulling hair) | SDQ Parent Total Score 14-16 |
|  | 2 | Moderately intense outbursts, 2-4 times a week | SDQ Parent Total Score 17-19 |
|  | 3 | Multiple outbursts daily, physical aggression towards self or others, may require 1-2 medications (stimulants, antipsychotics etc.) | SDQ Parent Total Score 20-22 |
|  | 4 | Multiple outbursts daily, severe aggression resulting in multiple injuries, may require more than 2 medications | SDQ Parent Total Score >22 |
| Sleep disturbance | 0 | Normal sleep pattern |  |
|  | 1 | Present, some disruption, No medications needed | For age 6-15, SDSC T-score <55; For age 2-6, CSWS mean total score >3.5 |
|  | 2 | Significant disruption, requiring single sleep medication | For age 6-15, SDSC T-score 55-70; For age 2-6, CSWS mean total score 3.0-3.5 |
|  | 3 | Significant disruption, requiring more than one sleep medication | For age 6-15, SDSC T-score >70; For age 2-6, CSWS mean total score <3 |
| *Total Neurodevelopment* | Max = 24 | |  |
| **Systemic features** |  |  |  |
| Palate | 0 | Intact palate, high arched, bifid uvula |  |
|  | 2 | Cleft palate | Includes submucous cleft palate |
| Sialorrhea | 0 | Normal drooling |  |
|  | 1 | Excessive drooling after 2 years of age with no intervention needed |  |
|  | 2 | Excessive drooling after 2 years of age requiring intervention | Interventions include medications (Glygopyrrolate, scopolamine, Botox) or surgery |
| Strabismus | 0 | Not present |  |
|  | 1 | Present, no intervention needed by 2 years of age |  |
|  | 2 | Present and patching or surgery needed at any age | Interventions include patching, eye drops, or surgery |
| Scoliosis | 0 | Not present or mild, only noticed by X-rays, no interventions needed | <10˚ |
|  | 1 | Moderate, noticed by both clinical examination and X-rays, needed brace | 10˚ to <40˚ |
|  | 2 | Severe, clearly visible by examination or spine surgery needed | >40˚ or surgical intervention needed |
| Muscle tone (past or present, first 5 years) | 0 | Normal |  |
|  | 1 | Tone mildly abnormal |  |
|  | 2 | Severe or generalized hypotonia, dystonia or hypertonia |  |
| Bone health | 0 | Normal, no previous fractures | BMD (z-score) >-1.5 |
|  | 1 | Only explainable traumatic fractures | BMD (z-score): -1.5 to -1.9 |
|  | 2 | One or multiple unexplained fractures, not needing bone resorption inhibitors | BMD (z-score): -2.0 to -2.4 |
|  | 3 | One or multiple unexplained fractures, needing bone resorption inhibitors | BMD (z-score): ≤-2.5 |
| Somatic growth and feeding | 0 | No growth failure |  |
|  | 1 | Weight between -1 and -2 SD (3% <w< 25%) or Weight for length/BMI <2SD (<3%) |  |
|  | 2 | Weight below 2SD (<3%) |  |
|  | 3 | Gastrostomy tube (G-tube) needed regardless of growth |  |
| Epilepsy/Seizures | 0 | No clinical seizures |  |
|  | 1 | Previous suspected/confirmed seizures or current well-controlled (<1x month) with a single antiepileptic | Includes febrile seizures |
|  | 2 | Current uncontrolled (>1x/month) seizures or needs more than 1 antiepileptic medication to control |  |
| Dental | 0 | No morphological dental abnormalities |  |
|  | 5 | Any of the following clinical or radiographic findings, 1 point each with a maximum of 5 points: Macrodontia, excessive crowding, delayed eruption, abnormal dental shape, history of dental trauma, missing mandibular 2^nd^ bicuspids, missing maxillary 2^nd^ bicuspids, lack of 6-year molars root development, severely rotated teeth |  |
| *Total Systemic* | Max = 23 | |  |
| **Total Severity Score (Neurodevelopmental + systemic)** | Max = 47 | |  |

^a^Vineland 70-85 parent or teacher, ABAS-3 composite 70-85, DAS-2 composite 50-70, DAYC-2 50-70, TELD-3 50-70, TACL-4 centile 1-5, BDI-2 Cognitive 50-70, DQ 50-70, DP-3 50-70, Mullen 50-70, Kaufman 50-70.

^b^Vineland <70, ABAS-3 composite <70, DAS-2 composite <50, DAYC-2 <50, TELD-3 <50, TACL-4 centile <1, BDI-2 Cognitive <50, DQ<50, DP-3 <50, Mullen <50, Kaufman <50, Batelle< 50% delay.

Abbreviations: BMD, bone mineral density; CSWS, Children’s Sleep Wake Scale; MLU, mean length of utterance; SDQ: strengths and difficulties questionnaire; SDSC, sleep disturbance scale for children.

**Table S2.** Demographic and phenotypic features of 164 individuals with *SATB2*-associated syndrome

| **Characteristic** | **All patients (n = 164)** |
| --- | --- |
| **Demographics** |  |
| Male  Female | 86 (52.4%)  78 (47.6) |
| Mean age, years | 10.8±6.9 |
|  |  |
| **Molecular mechanism** |  |
| Missense | 45 (27.4%) |
| CUT1 | 30 (18.3%) |
| CUT2 | 5 (3.0%) |
| HOX | 5 (3.0%) |
| ULD | 4 (2.4%) |
| No domain | 1 (0.6%) |
| Arg389Cys | 10 (6.1%) |
|  |  |
| Null | 93 (56.7%) |
| Frameshift | 33 (20.1%) |
| Nonsense | 33 (20.1%) |
| Intragenenic deletion | 17 (10.4%) |
| Splicing | 10 (6.1%) |
|  |  |
| Chromosomal | 25 |
| Deletion <6Mb | 12 |
| Deletion >6Mb | 10 |
| Duplication | 3 |
|  |  |
| Intronic | 1 (%) |

**Table S3.** Multiple linear regression for SAS severity score by variant groups, adjusted for age group and sex.

| **Mutation Category** | **n** | **Mean** | **95% CI** | **Coef.** | **95% CI** | ***P*-value** |
| --- | --- | --- | --- | --- | --- | --- |
| Missense | 93 | 19.01 | 17.18, 20.84 | Ref | Ref | Ref |
| Null | 45 | 20.34 | 19.04, 21.64 | 1.33 | -0.83, 3.5 | 0.226 |
| Chromosomal | 25 | 20.37 | 17.94, 22.81 | 1.36 | -1.64, 4.37 | 0.3721 |
|  |  |  |  |  |  |  |
| Molecular Subcategory* |  |  |  |  |  |  |
| Missense HOX | 5 | 14.44 | 9.27, 19.61 | Ref | Ref | Ref |
| Missense Other | 5 | 15.69 | 10.49, 20.88 | 1.25 | -6.01, 8.5 | 0.7346 |
| Missense CUT2 | 5 | 16.94 | 11.8, 22.08 | 2.5 | -4.75, 9.75 | 0.4965 |
| Null and <aa350 | 38 | 18.21 | 16.32, 20.1 | 3.77 | -1.72, 9.26 | 0.1765 |
| Chromosomal <6Mb | 12 | 19.08 | 15.73, 22.43 | 4.64 | -1.44, 10.72 | 0.1336 |
| MissenseCUT1Other | 20 | 19.79 | 17.21, 22.37 | 5.35 | -0.45, 11.16 | 0.0705 |
| Splice | 7 | 20.24 | 15.94, 24.55 | 5.81 | -0.92, 12.53 | 0.0903 |
| Null and >aa350 | 31 | 21.52 | 19.35, 23.69 | 7.08 | 1.56, 12.6 | 0.0123 |
| Missense Arg389Cys | 10 | 21.87 | 18.18, 25.57 | 7.43 | 1.09, 13.78 | 0.022 |
| Null Intragenic Del | 17 | 22.33 | 19.51, 25.14 | 7.89 | 1.96, 13.81 | 0.0094 |
| Chromosomal >6Mb | 10 | 23.73 | 20.08, 27.39 | 9.3 | 2.94, 15.65 | 0.0044 |

*Excluding one individual with an intronic variant and 3 individuals with whole gene duplications.

Abbreviations: aa, amino acid; CI, confidence interval; Coef, coefficient; Ref, reference.

**Table S4.** Multiple linear regression for neurodevelopmental score by variant groups, adjusted for age group and sex.

| **Mutation Category** | **n** | **Mean** | **95% CI** | **Coef.** | **95% CI** | ***P*-value** |
| --- | --- | --- | --- | --- | --- | --- |
| Missense | 93 | 12.04 | 10.75, 13.34 | Ref | Ref | Ref |
| Null | 45 | 12.63 | 11.71, 13.55 | 0.59 | -0.95, 2.12 | 0.4513 |
| Chromosomal | 25 | 12.67 | 10.94, 14.39 | 0.63 | -1.5, 2.75 | 0.5625 |
|  |  |  |  |  |  |  |
| Molecular Subcategory* |  |  |  |  |  |  |
| Missense Other | 5 | 8.77 | 5.02, 12.51 | -1.82 | -7.05, 3.41 | 0.4922 |
| Missense CUT2 | 5 | 8.97 | 5.27, 12.68 | -1.62 | -6.84, 3.61 | 0.5421 |
| Missense HOX | 5 | 10.59 | 6.86, 14.32 | Ref | Ref | Ref |
| Null and <aa350 | 38 | 11.54 | 10.18, 12.9 | 0.95 | -3.01, 4.91 | 0.6358 |
| Chromosomal <6Mb | 12 | 11.86 | 9.44, 14.27 | 1.27 | -3.12, 5.65 | 0.5693 |
| Missense CUT1 other | 20 | 12.91 | 11.05, 14.77 | 2.32 | -1.87, 6.5 | 0.2755 |
| Null and >=aa350 | 31 | 13.08 | 11.52, 14.65 | 2.49 | -1.49, 6.47 | 0.2176 |
| Null intragenic deletion | 17 | 13.26 | 11.23, 15.29 | 2.67 | -1.6, 6.94 | 0.2186 |
| Splice | 7 | 13.39 | 10.29, 16.5 | 2.8 | -2.05, 7.65 | 0.2554 |
| Arg389Cys | 10 | 13.64 | 10.97, 16.3 | 3.05 | -1.53, 7.62 | 0.1902 |
| Chromosomal >6Mb | 10 | 15.02 | 12.38, 17.65 | 4.42 | -0.16, 9.01 | 0.0583 |

*Excluding one individual with an intronic variant and 3 individuals with whole gene duplications.

Abbreviations: aa, amino acid; CI, confidence interval; Coef, coefficient; Ref, reference.

**Table S5.** Multiple linear regression for systemic score by variant groups, adjusted for age group and sex.

| **Mutation Category** | **n** | **Mean** | **95% CI** | **Coef.** | **95% CI** | ***P*-value** |
| --- | --- | --- | --- | --- | --- | --- |
| Missense | 93 | 6.97 | 6.11, 7.83 | Ref | Ref | Ref |
| Null | 45 | 7.71 | 7.10, 8.33 | 0.75 | -0.27, 1.77 | 0.4513 |
| Chromosomal | 25 | 7.70 | 6.56, 8.85 | 0.74 | -0.68, 2.15 | 0.3064 |
|  |  |  |  |  |  |  |
| Molecular Subcategory* |  |  |  |  |  |  |
| Missense HOX | 5 | 3.85 | 1.43, 6.26 | Ref | Ref | Ref |
| Null and <aa350 | 38 | 6.67 | 5.79, 7.55 | 2.82 | 0.26, 5.39 | 0.0314 |
| Splice | 7 | 6.85 | 4.84, 8.86 | 3 | -0.14, 6.15 | 0.0612 |
| Missense CUT1 other | 20 | 6.88 | 5.68, 8.09 | 3.03 | 0.32, 5.75 | 0.0287 |
| Missense Other | 5 | 6.92 | 4.49, 9.34 | 3.07 | -0.32, 6.46 | 0.0758 |
| Chromosomal <6Mb | 12 | 7.22 | 5.66, 8.79 | 3.38 | 0.53, 6.22 | 0.0203 |
| Missense CUT2 | 5 | 7.96 | 5.56, 10.37 | 4.12 | 0.73, 7.51 | 0.0176 |
| Arg389Cys | 10 | 8.23 | 6.51, 9.96 | 4.39 | 1.42, 7.35 | 0.004 |
| Null and >=aa350 | 31 | 8.44 | 7.42, 9.45 | 4.59 | 2.01, 7.17 | 0.0006 |
| Chromosomal >6Mb | 10 | 8.72 | 7.01, 10.43 | 4.87 | 1.9, 7.84 | 0.0015 |
| Null intragenic deletion | 17 | 9.06 | 7.75, 10.38 | 5.22 | 2.45, 7.99 | 0.0003 |

*Excluding one individual with an intronic variant and 3 individuals with whole gene duplications.

Abbreviations: aa, amino acid; CI, confidence interval; Coef, coefficient; Ref, reference.

**Table S6.** Multiple linear regression for adaptive/cognition category score by variant groups, adjusted for age group and sex.

| **Mutation Category** | **n** | **Mean** | **95% CI** | **Coef.** | **95% CI** | ***P*-value** |
| --- | --- | --- | --- | --- | --- | --- |
| Missense | 93 | 4.21 | [3.80, 4.61] | Ref | Ref | Ref |
| Null | 45 | 4.15 | [3.87, 4.44] | -0.05 | [-0.53, 0.43] | 0.8305 |
| Chromosomal | 25 | 4.55 | [4.01, 5.09] | 0.34 | [0.32, 1.01] | 0.3113 |
|  |  |  |  |  |  |  |
| Molecular Subcategory* |  |  |  |  |  |  |
| Missense Other | 5 | 3.23 | 2.02, 4.43 | -1.01 | -2.69, 0.67 | 0.2356 |
| Missense CUT2 | 5 | 3.28 | 2.09, 4.46 | -0.96 | -2.64, 0.72 | 0.2592 |
| Null and <aa350 | 38 | 3.81 | 3.37, 4.25 | -0.43 | -1.7, 0.84 | 0.508 |
| Null and >=aa350 | 31 | 4.21 | 3.7, 4.71 | -0.03 | -1.31, 1.25 | 0.964 |
| Missense HOX | 5 | 4.24 | 3.04, 5.43 | Ref | Ref | Ref |
| Missense CUT1 other | 20 | 4.37 | 3.77, 4.96 | 0.13 | -1.21, 1.47 | 0.8473 |
| Chromosomal <6Mb | 12 | 4.51 | 3.73, 5.28 | 0.27 | -1.14, 1.68 | 0.7058 |
| Null intragenic deletion | 17 | 4.57 | 3.92, 5.22 | 0.33 | -1.04, 1.7 | 0.6329 |
| Chromosomal >6Mb | 10 | 4.60 | 3.75, 5.44 | 0.36 | -1.11, 1.83 | 0.6275 |
| Splice | 7 | 4.66 | 3.66, 5.65 | 0.42 | -1.14, 1.98 | 0.5951 |
| Arg389Cys | 10 | 4.78 | 3.93, 5.64 | 0.55 | -0.92, 2.02 | 0.4624 |

*Excluding one individual with an intronic variant and 3 individuals with whole gene duplications.

Abbreviations: aa, amino acid; CI, confidence interval; Coef, coefficient; Ref, reference.

**Table S7.** Multiple linear regression for verbal category score by variant groups, adjusted for age group and sex.

| **Mutation Category** | **n** | **Mean** | **95% CI** | **Coef.** | **95% CI** | ***P*-value** |
| --- | --- | --- | --- | --- | --- | --- |
| Missense | 93 | 2.19 | 1.79, 2.59 | Ref | Ref | Ref |
| Null | 45 | 2.41 | 2.12, 2.69 | 0.21 | -0.25, 0.68 | 0.3666 |
| Chromosomal | 25 | 2.09 | 1.56, 2.62 | -0.1 | -0.75, 0.55 | 0.7619 |
|  |  |  |  |  |  |  |
| Molecular Subcategory* |  |  |  |  |  |  |
| Missense HOX | 5 | 1.17 | 0.03, 2.31 | Ref | Ref | Ref |
| Missense CUT2 | 5 | 1.21 | 0.08, 2.34 | 0.04 | -1.55, 1.64 | 0.9578 |
| Missense Other | 5 | 1.38 | 0.24, 2.52 | 0.21 | -1.39, 1.8 | 0.797 |
| Chromosomal <6Mb | 12 | 2.06 | 1.33, 2.8 | 0.89 | -0.45, 2.23 | 0.19 |
| Splice | 7 | 2.06 | 1.12, 3.01 | 0.89 | -0.59, 2.37 | 0.2358 |
| Null and <aa350 | 38 | 2.31 | 1.9, 2.73 | 1.14 | -0.06, 2.35 | 0.0633 |
| Missense CUT1 other | 20 | 2.45 | 1.88, 3.01 | 1.28 | 0, 2.55 | 0.0502 |
| Null intragenic deletion | 17 | 2.47 | 1.85, 3.09 | 1.29 | -0.01, 2.6 | 0.0515 |
| Null and >=aa350 | 31 | 2.49 | 2.02, 2.97 | 1.32 | 0.11, 2.54 | 0.033 |
| Chromosomal >6Mb | 10 | 2.62 | 1.81, 3.42 | 1.45 | 0.05, 2.85 | 0.0426 |
| Arg389Cys | 10 | 2.98 | 2.16, 3.79 | 1.81 | 0.41, 3.2 | 0.0116 |

*Excluding one individual with an intronic variant and 3 individuals with whole gene duplications.

Abbreviations: aa, amino acid; CI, confidence interval; Coef, coefficient; Ref, reference.

**Table S8.** Multiple linear regression for expressive category score by variant groups, adjusted for age group and sex.

| **Mutation Category** | **n** | **Mean** | **95% CI** | **Coef.** | **95% CI** | ***P*-value** |
| --- | --- | --- | --- | --- | --- | --- |
| Missense | 93 | 2.26 | 1.83, 2.69 | Ref | Ref | Ref |
| Null | 45 | 2.36 | 2.05, 2.67 | 0.1 | -0.42, 0.61 | 0.7113 |
| Chromosomal | 25 | 2.57 | 2.00, 3.15 | 0.31 | -0.40, 1.02 | 0.3927 |
|  |  |  |  |  |  |  |
| Molecular Subcategory* |  |  |  |  |  |  |
| Missense CUT2 | 5 | 1.50 | 0.23, 2.77 | -0.14 | -1.94, 1.65 | 0.8747 |
| Missense HOX | 5 | 1.65 | 0.37, 2.93 | Ref | Ref | Ref |
| Chromosomal <6Mb | 12 | 1.99 | 1.16, 2.82 | 0.35 | -1.16, 1.85 | 0.6509 |
| Missense Other | 5 | 2.09 | 0.8, 3.37 | 0.44 | -1.35, 2.23 | 0.6289 |
| Arg389Cys | 10 | 2.17 | 1.26, 3.08 | 0.52 | -1.05, 2.09 | 0.5109 |
| Null and <aa350 | 38 | 2.20 | 1.74, 2.67 | 0.56 | -0.8, 1.91 | 0.4197 |
| Null intragenic deletion | 17 | 2.26 | 1.57, 2.96 | 0.62 | -0.85, 2.08 | 0.4072 |
| Null and >=aa350 | 31 | 2.46 | 1.92, 3 | 0.82 | -0.55, 2.18 | 0.2396 |
| Splice | 7 | 2.54 | 1.48, 3.61 | 0.9 | -0.77, 2.56 | 0.2891 |
| Missense CUT1 other | 20 | 2.62 | 1.98, 3.26 | 0.97 | -0.46, 2.41 | 0.1829 |
| Chromosomal >6Mb | 10 | 3.47 | 2.57, 4.37 | 1.82 | 0.25, 3.39 | 0.0233 |

*Excluding one individual with an intronic variant and 3 individuals with whole gene duplications.

Abbreviations: aa, amino acid; CI, confidence interval; Coef, coefficient; Ref, reference.

**Table S9.** Multiple linear regression for ambulation category score by variant groups, adjusted for age group and sex.

| **Mutation Category** | **n** | **Mean** | **95% CI** | **Coef.** | **95% CI** | ***P*-value** |
| --- | --- | --- | --- | --- | --- | --- |
| Missense | 93 | 1.20 | 0.96, 1.43 | Ref | Ref | Ref |
| Null | 45 | 1.20 | 1.03, 1.36 | 0 | -0.28, 0.28 | 0.998 |
| Chromosomal | 25 | 1.28 | 0.97, 1.59 | 0.08 | -0.3, 0.46 | 0.6804 |
|  |  |  |  |  |  |  |
| Molecular Subcategory* |  |  |  |  |  |  |
| Missense HOX | 5 | 0.89 | 0.22, 1.56 | Ref | Ref | Ref |
| Chromosomal <6Mb | 12 | 0.94 | 0.5, 1.37 | 0.04 | -0.75, 0.83 | 0.9138 |
| Null and <aa350 | 38 | 0.94 | 0.7, 1.19 | 0.05 | -0.66, 0.76 | 0.891 |
| Splice | 7 | 1.02 | 0.46, 1.58 | 0.12 | -0.75, 1 | 0.7779 |
| Arg389Cys | 10 | 1.14 | 0.66, 1.62 | 0.25 | -0.58, 1.07 | 0.5556 |
| Missense CUT1 other | 20 | 1.20 | 0.87, 1.53 | 0.31 | -0.45, 1.06 | 0.4213 |
| Missense Other | 5 | 1.28 | 0.6, 1.95 | 0.38 | -0.56, 1.33 | 0.4199 |
| Null and >=aa350 | 31 | 1.36 | 1.08, 1.64 | 0.46 | -0.25, 1.18 | 0.2012 |
| Missense CUT2 | 5 | 1.39 | 0.72, 2.06 | 0.5 | -0.44, 1.44 | 0.2987 |
| Null intragenic deletion | 17 | 1.47 | 1.1, 1.83 | 0.57 | -0.2, 1.34 | 0.1427 |
| Chromosomal >6Mb | 10 | 1.86 | 1.39, 2.34 | 0.97 | 0.15, 1.8 | 0.0212 |

*Excluding one individual with an intronic variant and 3 individuals with whole gene duplications.

Abbreviations: aa, amino acid; CI, confidence interval; Coef, coefficient; Ref, reference.

**Table S10.** Multiple linear regression for behavior category score by variant groups, adjusted for age group and sex.

| **Mutation Category** | **n** | **Mean** | **95% CI** | **Coef.** | **95% CI** | ***P*-value** |
| --- | --- | --- | --- | --- | --- | --- |
| Missense | 93 | 1.37 | 1.03, 1.71 | Ref | Ref | Ref |
| Null | 45 | 1.46 | 1.22, 1.7 | 0.09 | -0.31, 0.49 | 0.6436 |
| Chromosomal | 25 | 1.24 | 0.79, 1.69 | -0.13 | -0.68, 0.43 | 0.6462 |
|  |  |  |  |  |  |  |
| Molecular Subcategory* |  |  |  |  |  |  |
| Missense Other | 5 | 0.39 | -0.61, 1.4 | -1.6 | -3, -0.19 | 0.0261 |
| Missense CUT1 other | 20 | 1.20 | 0.7, 1.7 | -0.78 | -1.91, 0.34 | 0.1695 |
| Null and <aa350 | 38 | 1.28 | 0.92, 1.65 | -0.71 | -1.77, 0.35 | 0.19 |
| Chromosomal <6Mb | 12 | 1.35 | 0.7, 2 | -0.64 | -1.82, 0.54 | 0.2853 |
| Chromosomal >6Mb | 10 | 1.35 | 0.65, 2.06 | -0.63 | -1.86, 0.6 | 0.3101 |
| Null and >=aa350 | 31 | 1.49 | 1.07, 1.91 | -0.5 | -1.56, 0.57 | 0.3599 |
| Splice | 7 | 1.54 | 0.71, 2.37 | -0.45 | -1.75, 0.86 | 0.499 |
| Missense CUT2 | 5 | 1.59 | 0.59, 2.58 | -0.4 | -1.8, 1 | 0.5747 |
| Arg389Cys | 10 | 1.70 | 0.99, 2.42 | -0.28 | -1.51, 0.94 | 0.6485 |
| Null intragenic deletion | 17 | 1.71 | 1.17, 2.26 | -0.28 | -1.42, 0.87 | 0.6349 |
| Missense HOX | 5 | 1.99 | 0.99, 2.99 | Ref | Ref | Ref |

*Excluding one individual with an intronic variant and 3 individuals with whole gene duplications.

Abbreviations: aa, amino acid; CI, confidence interval; Coef, coefficient; Ref, reference.

**Table S11.** Multiple linear regression for sleep category score by variant groups, adjusted for age group and sex.

| **Mutation Category** | **n** | **Mean** | **95% CI** | **Coef.** | **95% CI** | ***P*-value** |
| --- | --- | --- | --- | --- | --- | --- |
| Missense | 93 | 0.94 | 0.62, 1.27 | Ref | Ref | Ref |
| Null | 45 | 1.07 | 0.84, 1.3 | 0.12 | -0.26, 0.51 | 0.5289 |
| Chromosomal | 25 | 0.92 | 0.49, 1.36 | -0.02 | -0.56, 0.51 | 0.9344 |
|  |  |  |  |  |  |  |
| Molecular Subcategory* |  |  |  |  |  |  |
| Missense Other | 5 | 0.46 | -0.52, 1.45 | -0.2 | -1.58, 1.18 | 0.7736 |
| Missense HOX | 5 | 0.66 | -0.32, 1.65 | Ref | Ref | Ref |
| Null intragenic deletion | 17 | 0.80 | 0.26, 1.33 | 0.13 | -0.99, 1.26 | 0.8148 |
| Arg389Cys | 10 | 0.89 | 0.19, 1.59 | 0.23 | -0.98, 1.43 | 0.7127 |
| Missense CUT2 | 5 | 0.99 | 0.01, 1.96 | 0.32 | -1.05, 1.7 | 0.6449 |
| Null and <aa350 | 38 | 1.01 | 0.65, 1.36 | 0.34 | -0.7, 1.38 | 0.5192 |
| Chromosomal <6Mb | 12 | 1.02 | 0.38, 1.65 | 0.35 | -0.8, 1.51 | 0.5489 |
| Chromosomal >6Mb | 10 | 1.07 | 0.37, 1.76 | 0.4 | -0.8, 1.61 | 0.5094 |
| Missense CUT1 other | 20 | 1.09 | 0.6, 1.58 | 0.43 | -0.67, 1.53 | 0.4426 |
| Null and >=aa350 | 31 | 1.11 | 0.7, 1.52 | 0.45 | -0.6, 1.49 | 0.4021 |
| Splice | 7 | 1.57 | 0.75, 2.39 | 0.9 | -0.37, 2.18 | 0.164 |

*Excluding one individual with an intronic variant and 3 individuals with whole gene duplications.

Abbreviations: aa, amino acid; CI, confidence interval; Coef, coefficient; Ref, reference.

**Table S12.** Multiple linear regression for palate category score by variant groups, adjusted for age group and sex.

| **Mutation Category** | **n** | **Mean** | **95% CI** | **Coef.** | **95% CI** | ***P*-value** |
| --- | --- | --- | --- | --- | --- | --- |
| Missense | 93 | 0.35 | 0.06, 0.63 | Ref | Ref | Ref |
| Null | 45 | 0.96 | 0.75, 1.16 | 0.61 | 0.27, 0.95 | 0.0005 |
| Chromosomal | 25 | 1.02 | 0.64, 1.4 | 0.67 | 0.20, 1.14 | 0.0054 |
|  |  |  |  |  |  |  |
| Molecular Subcategory* |  |  |  |  |  |  |
| Missense CUT1 other | 20 | 0.17 | -0.25, 0.59 | -0.24 | -1.18, 0.69 | 0.607 |
| Missense Other | 5 | 0.41 | -0.43, 1.25 | 0 | -1.18, 1.17 | 0.9944 |
| Missense HOX | 5 | 0.42 | -0.42, 1.25 | Ref | Ref | Ref |
| Arg389Cys | 10 | 0.46 | -0.13, 1.06 | 0.05 | -0.98, 1.07 | 0.9271 |
| Missense CUT2 | 5 | 0.50 | -0.33, 1.33 | 0.08 | -1.09, 1.25 | 0.8927 |
| Splice | 7 | 0.55 | -0.14, 1.25 | 0.14 | -0.95, 1.22 | 0.806 |
| Null and >=aa350 | 31 | 0.69 | 0.34, 1.05 | 0.28 | -0.61, 1.17 | 0.5378 |
| Chromosomal <6Mb | 12 | 0.73 | 0.19, 1.27 | 0.31 | -0.67, 1.29 | 0.5313 |
| Null and <aa350 | 38 | 1.03 | 0.72, 1.33 | 0.61 | -0.28, 1.5 | 0.1762 |
| Null intragenic deletion | 17 | 1.33 | 0.87, 1.78 | 0.91 | -0.05, 1.87 | 0.062 |
| Chromosomal >6Mb | 10 | 1.67 | 1.08, 2.26 | 1.25 | 0.22, 2.28 | 0.0173 |

*Excluding one individual with an intronic variant and 3 individuals with whole gene duplications.

Abbreviations: aa, amino acid; CI, confidence interval; Coef, coefficient; Ref, reference.

**Table S13.** Multiple linear regression for sialorrhea category score by variant groups, adjusted for age group and sex.

| **Mutation Category** | **n** | **Mean** | **95% CI** | **Coef.** | **95% CI** | ***P*-value** |
| --- | --- | --- | --- | --- | --- | --- |
| Missense | 93 | 0.85 | 0.66, 1.05 | Ref | Ref | Ref |
| Null | 45 | 0.88 | 0.74, 1.01 | 0.02 | -0.21, 0.25 | 0.8476 |
| Chromosomal | 25 | 0.66 | 0.41, 0.92 | -0.19 | -0.51, 0.13 | 0.2428 |
|  |  |  |  |  |  |  |
| Molecular Subcategory* |  |  |  |  |  |  |
| Missense Other | 5 | 0.34 | -0.22, 0.9 | -0.15 | -0.92, 0.63 | 0.7126 |
| Missense CUT2 | 5 | 0.36 | -0.19, 0.92 | -0.12 | -0.9, 0.66 | 0.7599 |
| Missense HOX | 5 | 0.49 | -0.07, 1.04 | Ref | Ref | Ref |
| Splice | 7 | 0.57 | 0.11, 1.03 | 0.08 | -0.64, 0.81 | 0.8173 |
| Chromosomal <6Mb | 12 | 0.69 | 0.33, 1.05 | 0.21 | -0.45, 0.86 | 0.5325 |
| Chromosomal >6Mb | 10 | 0.71 | 0.32, 1.11 | 0.23 | -0.45, 0.91 | 0.5082 |
| Null and <aa350 | 38 | 0.79 | 0.58, 0.99 | 0.3 | -0.29, 0.89 | 0.3138 |
| Missense CUT1 other | 20 | 0.93 | 0.65, 1.2 | 0.44 | -0.18, 1.06 | 0.164 |
| Null and >=aa350 | 31 | 0.97 | 0.74, 1.21 | 0.49 | -0.1, 1.08 | 0.105 |
| Null intragenic deletion | 17 | 1.04 | 0.74, 1.34 | 0.56 | -0.08, 1.19 | 0.0859 |
| Arg389Cys | 10 | 1.41 | 1.02, 1.81 | 0.93 | 0.25, 1.61 | 0.0079 |

*Excluding one individual with an intronic variant and 3 individuals with whole gene duplications.

Abbreviations: aa, amino acid; CI, confidence interval; Coef, coefficient; Ref, reference.

**Table S14.** Multiple linear regression for strabismus category score by variant groups, adjusted for age group and sex.

| **Mutation Category** | **n** | **Mean** | **95% CI** | **Coef.** | **95% CI** | ***P*-value** |
| --- | --- | --- | --- | --- | --- | --- |
| Missense | 93 | 0.63 | 0.40, 0.86 | Ref | Ref | Ref |
| Null | 45 | 0.59 | 0.43, 0.76 | -0.04 | -0.31, 0.24 | 0.7937 |
| Chromosomal | 25 | 0.55 | 0.24, 0.86 | -0.08 | -0.46, 0.3 | 0.6836 |
|  |  |  |  |  |  |  |
| Molecular Subcategory* |  |  |  |  |  |  |
| Missense HOX | 5 | 0.33 | -0.36, 1.02 | Ref | Ref | Ref |
| Null intragenic deletion | 17 | 0.39 | 0.01, 0.76 | 0.06 | -0.73, 0.85 | 0.8805 |
| Splice | 7 | 0.44 | -0.14, 1.01 | 0.11 | -0.79, 1.01 | 0.8066 |
| Missense Other | 5 | 0.46 | -0.23, 1.16 | 0.14 | -0.83, 1.11 | 0.7811 |
| Null and <aa350 | 38 | 0.52 | 0.27, 0.77 | 0.19 | -0.54, 0.93 | 0.603 |
| Chromosomal >6Mb | 10 | 0.54 | 0.05, 1.03 | 0.21 | -0.64, 1.07 | 0.6182 |
| Chromosomal <6Mb | 12 | 0.63 | 0.18, 1.08 | 0.3 | -0.51, 1.12 | 0.4616 |
| Missense CUT1 other | 20 | 0.63 | 0.28, 0.97 | 0.3 | -0.48, 1.08 | 0.4481 |
| Missense CUT2 | 5 | 0.75 | 0.06, 1.44 | 0.42 | -0.55, 1.39 | 0.3901 |
| Arg389Cys | 10 | 0.82 | 0.33, 1.32 | 0.5 | -0.35, 1.34 | 0.2508 |
| Null and >=aa350 | 31 | 0.84 | 0.55, 1.13 | 0.51 | -0.22, 1.25 | 0.1709 |

*Excluding one individual with an intronic variant and 3 individuals with whole gene duplications.

Abbreviations: aa, amino acid; CI, confidence interval; Coef, coefficient; Ref, reference.

**Table S15.** Multiple linear regression for scoliosis category score by variant groups, adjusted for age group and sex.

| **Mutation Category** | **n** | **Mean** | **95% CI** | **Coef.** | **95% CI** | ***P* value** |
| --- | --- | --- | --- | --- | --- | --- |
| Missense | 93 | 0.23 | 0.12, 0.33 | Ref | Ref | Ref |
| Null | 45 | 0.10 | 0.02, 0.18 | -0.13 | -0.26, 0 | 0.0525 |
| Chromosomal | 25 | 0.10 | -0.04, 0.25 | -0.12 | -0.3, 0.06 | 0.1853 |
|  |  |  |  |  |  |  |
| Molecular Subcategory* |  |  |  |  |  |  |
| Splice | 7 | 0.00 | -0.27, 0.28 | -0.21 | -0.64, 0.22 | 0.3434 |
| Null intragenic deletion | 17 | 0.01 | -0.17, 0.19 | -0.2 | -0.57, 0.18 | 0.3082 |
| Chromosomal <6Mb | 12 | 0.02 | -0.19, 0.24 | -0.18 | -0.57, 0.2 | 0.3508 |
| Missense Other | 5 | 0.06 | -0.27, 0.39 | -0.15 | -0.61, 0.31 | 0.519 |
| Null and <aa350 | 38 | 0.10 | -0.02, 0.22 | -0.11 | -0.46, 0.24 | 0.5404 |
| Null and >=aa350 | 31 | 0.16 | 0.02, 0.3 | -0.05 | -0.4, 0.31 | 0.7947 |
| Chromosomal >6Mb | 10 | 0.19 | -0.04, 0.43 | -0.01 | -0.42, 0.39 | 0.9438 |
| Missense HOX | 5 | 0.21 | -0.12, 0.54 | Ref | Ref | Ref |
| Arg389Cys | 10 | 0.22 | -0.01, 0.46 | 0.02 | -0.39, 0.42 | 0.9343 |
| Missense CUT1 other | 20 | 0.22 | 0.06, 0.38 | 0.01 | -0.36, 0.38 | 0.9444 |
| Missense CUT2 | 5 | 0.42 | 0.1, 0.75 | 0.22 | -0.25, 0.68 | 0.3566 |

*Excluding one individual with an intronic variant and 3 individuals with whole gene duplications.

Abbreviations: aa, amino acid; CI, confidence interval; Coef, coefficient; Ref, reference.

**Table S16.** Multiple linear regression for muscle tone category score by variant groups, adjusted for age group and sex.

| **Mutation Category** | **n** | **Mean** | **95% CI** | **Coef.** | **95% CI** | ***P*-value** |
| --- | --- | --- | --- | --- | --- | --- |
| Missense | 93 | 0.67 | 0.50, 0.73 | Ref | Ref | Ref |
| Null | 45 | 0.62 | 0.50, 0.73 | -0.05 | -0.24, 0.14 | 0.6078 |
| Chromosomal | 25 | 0.55 | 0.34, 0.77 | -0.11 | -0.37, 0.15 | 0.409 |
|  |  |  |  |  |  |  |
| Molecular Subcategory* |  |  |  |  |  |  |
| Splice | 7 | 0.30 | -0.09, 0.7 | -0.1 | -0.72, 0.52 | 0.7475 |
| Missense HOX | 5 | 0.40 | -0.07, 0.88 | Ref | Ref | Ref |
| Chromosomal <6Mb | 12 | 0.54 | 0.23, 0.85 | 0.14 | -0.42, 0.7 | 0.6253 |
| Missense CUT2 | 5 | 0.54 | 0.07, 1.01 | 0.14 | -0.53, 0.8 | 0.6856 |
| Null intragenic deletion | 17 | 0.55 | 0.29, 0.81 | 0.15 | -0.4, 0.69 | 0.595 |
| Null and <aa350 | 38 | 0.65 | 0.48, 0.83 | 0.25 | -0.25, 0.75 | 0.3246 |
| Missense CUT1 other | 20 | 0.65 | 0.41, 0.89 | 0.25 | -0.28, 0.78 | 0.3599 |
| Null and >=aa350 | 31 | 0.67 | 0.47, 0.87 | 0.27 | -0.24, 0.77 | 0.2949 |
| Arg389Cys | 10 | 0.73 | 0.39, 1.07 | 0.33 | -0.25, 0.91 | 0.262 |
| Chromosomal >6Mb | 10 | 0.77 | 0.44, 1.11 | 0.37 | -0.21, 0.95 | 0.2129 |
| Missense Other | 5 | 0.96 | 0.48, 1.44 | 0.56 | -0.11, 1.22 | 0.0993 |

*Excluding one individual with an intronic variant and 3 individuals with whole gene duplications.

Abbreviations: aa, amino acid; CI, confidence interval; Coef, coefficient; Ref, reference.

**Table S17.** Multiple linear regression for bone health category score by variant groups, adjusted for age group and sex.

| **Mutation Category** | **n** | **Mean** | **95% CI** | **Coef.** | **95% CI** | ***P*-value** |
| --- | --- | --- | --- | --- | --- | --- |
| Missense | 93 | 0.61 | 0.34, 0.88 | Ref | Ref | Ref |
| Null | 45 | 0.70 | 0.5, 0.89 | 0.09 | -0.24, 0.41 | 0.5941 |
| Chromosomal | 25 | 0.42 | 0.06, 0.79 | -0.19 | -0.64, 0.27 | 0.4176 |
|  |  |  |  |  |  |  |
| Molecular Subcategory* |  |  |  |  |  |  |
| Missense HOX | 5 | 0.02 | -0.79, 0.83 | Ref | Ref | Ref |
| Chromosomal >6Mb | 10 | 0.22 | -0.36, 0.79 | 0.19 | -0.8, 1.19 | 0.7022 |
| Missense Other | 5 | 0.44 | -0.37, 1.26 | 0.42 | -0.71, 1.56 | 0.4645 |
| Chromosomal <6Mb | 12 | 0.46 | -0.06, 0.99 | 0.44 | -0.51, 1.39 | 0.3597 |
| Splice | 7 | 0.55 | -0.12, 1.22 | 0.53 | -0.53, 1.58 | 0.3251 |
| Null and <aa350 | 38 | 0.56 | 0.26, 0.85 | 0.53 | -0.33, 1.39 | 0.2219 |
| Missense CUT1 other | 20 | 0.59 | 0.19, 0.99 | 0.57 | -0.34, 1.48 | 0.2197 |
| Arg389Cys | 10 | 0.77 | 0.19, 1.35 | 0.75 | -0.24, 1.74 | 0.1373 |
| Null and >=aa350 | 31 | 0.79 | 0.45, 1.13 | 0.77 | -0.1, 1.63 | 0.0817 |
| Null intragenic deletion | 17 | 0.98 | 0.54, 1.42 | 0.96 | 0.03, 1.88 | 0.0435 |
| Missense CUT2 | 5 | 1.25 | 0.45, 2.06 | 1.23 | 0.09, 2.36 | 0.0339 |

*Excluding one individual with an intronic variant and 3 individuals with whole gene duplications.

Abbreviations: aa, amino acid; CI, confidence interval; Coef, coefficient; Ref, reference.

**Table S18.** Multiple linear regression for growth and feeding category score by variant groups, adjusted for age group and sex.

| **Mutation Category** | **n** | **Mean** | **95% CI** | **Coef.** | **95% CI** | ***P*-value** |
| --- | --- | --- | --- | --- | --- | --- |
| Missense | 93 | 0.54 | 0.35, 0.76 | Ref | Ref | Ref |
| Null | 45 | 0.56 | 0.35, 0.76 | 0.02 | -0.32, 0.35 | 0.9195 |
| Chromosomal | 25 | 1.04 | 0.66, 1.42 | 0.5 | 0.03, 0.97 | 0.0364 |
|  |  |  |  |  |  |  |
| Molecular Subcategory* |  |  |  |  |  |  |
| Missense HOX | 5 | -0.04 | -0.86, 0.77 | Ref | Ref | Ref |
| Null and <aa350 | 38 | 0.21 | -0.08, 0.51 | 0.26 | -0.61, 1.12 | 0.5591 |
| Missense CUT1 other | 20 | 0.48 | 0.07, 0.89 | 0.52 | -0.39, 1.44 | 0.261 |
| Arg389Cys | 10 | 0.51 | -0.08, 1.09 | 0.55 | -0.45, 1.55 | 0.2801 |
| Chromosomal <6Mb | 12 | 0.60 | 0.07, 1.13 | 0.64 | -0.32, 1.6 | 0.1884 |
| Null intragenic deletion | 17 | 0.72 | 0.28, 1.17 | 0.77 | -0.17, 1.7 | 0.1078 |
| Splice | 7 | 0.73 | 0.05, 1.41 | 0.78 | -0.29, 1.84 | 0.1506 |
| Null and >=aa350 | 31 | 0.80 | 0.46, 1.15 | 0.84 | -0.03, 1.72 | 0.057 |
| Missense Other | 5 | 0.88 | 0.06, 1.7 | 0.92 | -0.22, 2.06 | 0.114 |
| Missense CUT2 | 5 | 0.95 | 0.14, 1.76 | 0.99 | -0.15, 2.13 | 0.0895 |
| Chromosomal >6Mb | 10 | 1.72 | 1.14, 2.3 | 1.76 | 0.76, 2.77 | 0.0007 |

*Excluding one individual with an intronic variant and 3 individuals with whole gene duplications.

Abbreviations: aa, amino acid; CI, confidence interval; Coef, coefficient; Ref, reference.

**Table S19.** Multiple linear regression for seizure category score by variant groups, adjusted for age group and sex.

| **Mutation Category** | **n** | **Mean** | **95% CI** | **Coef.** | **95% CI** | ***P*-value** |
| --- | --- | --- | --- | --- | --- | --- |
| Missense | 93 | 0.42 | 0.25, 0.59 | Ref | Ref | Ref |
| Null | 45 | 0.26 | 0.13, 0.38 | -0.17 | -0.37, 0.04 | 0.1085 |
| Chromosomal | 25 | 0.27 | 0.04, 0.5 | -0.15 | -0.43, 0.13 | 0.2958 |
|  |  |  |  |  |  |  |
| Molecular Subcategory* |  |  |  |  |  |  |
| Missense HOX | 5 | -0.03 | -0.53, 0.46 | Ref | Ref | Ref |
| Null and <aa350 | 38 | 0.05 | -0.13, 0.23 | 0.08 | -0.44, 0.61 | 0.7511 |
| Chromosomal <6Mb | 12 | 0.14 | -0.17, 0.46 | 0.18 | -0.4, 0.76 | 0.5424 |
| Splice | 7 | 0.15 | -0.26, 0.56 | 0.18 | -0.46, 0.82 | 0.5782 |
| Arg389Cys | 10 | 0.39 | 0.04, 0.75 | 0.43 | -0.18, 1.03 | 0.1638 |
| Missense CUT1 other | 20 | 0.41 | 0.17, 0.66 | 0.45 | -0.11, 1 | 0.1122 |
| Null intragenic deletion | 17 | 0.42 | 0.15, 0.69 | 0.45 | -0.11, 1.02 | 0.1153 |
| Null and >=aa350 | 31 | 0.44 | 0.23, 0.65 | 0.47 | -0.05, 1 | 0.0774 |
| Chromosomal >6Mb | 10 | 0.50 | 0.15, 0.85 | 0.53 | -0.07, 1.14 | 0.0852 |
| Missense Other | 5 | 0.59 | 0.09, 1.08 | 0.62 | -0.07, 1.31 | 0.0783 |
| Missense CUT2 | 5 | 0.78 | 0.29, 1.27 | 0.82 | 0.13, 1.51 | 0.021 |

*Excluding one individual with an intronic variant and 3 individuals with whole gene duplications.

Abbreviations: aa, amino acid; CI, confidence interval; Coef, coefficient; Ref, reference.

**Table S20.** Multiple linear regression for dental category score by variant groups, adjusted for age group and sex.

| **Mutation Category** | **n** | **Mean** | **95% CI** | **Coef.** | **95% CI** | ***P*-value** |
| --- | --- | --- | --- | --- | --- | --- |
| Missense | 93 | 2.68 | 2.25, 3.10 | Ref | Ref | Ref |
| Null | 45 | 3.06 | 2.76, 3.36 | 0.39 | -0.11, 0.89 | 0.1281 |
| Chromosomal | 25 | 3.07 | 2.51, 3.64 | 0.4 | -0.3, 1.09 | 0.2591 |
|  |  |  |  |  |  |  |
| Molecular Subcategory* |  |  |  |  |  |  |
| Missense HOX | 5 | 2.07 | 0.83, 3.3 | Ref | Ref | Ref |
| Chromosomal >6Mb | 10 | 2.40 | 1.53, 3.27 | 0.33 | -1.18, 1.85 | 0.6632 |
| Missense CUT2 | 5 | 2.41 | 1.19, 3.63 | 0.35 | -1.38, 2.07 | 0.6927 |
| Null and <aa350 | 38 | 2.77 | 2.32, 3.21 | 0.7 | -0.61, 2.01 | 0.2909 |
| Missense Other | 5 | 2.78 | 1.54, 4.01 | 0.71 | -1.01, 2.44 | 0.4164 |
| Missense CUT1 other | 20 | 2.81 | 2.19, 3.42 | 0.74 | -0.64, 2.12 | 0.2905 |
| Arg389Cys | 10 | 2.90 | 2.02, 3.78 | 0.84 | -0.67, 2.35 | 0.2745 |
| Null and >=aa350 | 31 | 3.07 | 2.55, 3.58 | 1 | -0.31, 2.31 | 0.1341 |
| Chromosomal <6Mb | 12 | 3.40 | 2.6, 4.2 | 1.34 | -0.11, 2.78 | 0.0702 |
| Splice | 7 | 3.56 | 2.54, 4.58 | 1.49 | -0.11, 3.1 | 0.0672 |
| Null intragenic deletion | 17 | 3.63 | 2.96, 4.3 | 1.56 | 0.15, 2.97 | 0.0299 |

*Excluding one individual with an intronic variant and 3 individuals with whole gene duplications.

Abbreviations: aa, amino acid; CI, confidence interval; Coef, coefficient; Ref, reference.
