# Supplementary material for: Quantitative Phenotype Morbidity Description of SATB2-Associated Syndrome
Source: Hum Mutat. 2023 Apr 26;2023:8200176. doi: 10.1155/2023/8200176 (PMC11918880; doi:10.1155/2023/8200176)
Supplement: Supplementary Materials — Figure S1: schematic representation of coding variants included in this study. Figures S2–S4: screenshots of the SATB2 portal and its different input modalities. Table S1: SATB2-associated syndrome severity score. Tables S2–S20: demographics and multiple linear regression models by mutation category for individual subcategories of the score. Supplementary File: individual score and molecular and demographic data for all individuals included in the study. [file 8200176.f1.zip › Supplementary File.pdf]

Mutation	Sub_mutation	SATB2 Registry ID	Age (years)	Sex
Missense	Missense	57	7	Female
	Missense	136	6	Male
	Missense	51	16	Female
	Missense	54	6	Male
	Missense	68	13	Male
	Missense	131	18	Male
	Missense	41	8	Female
	Missense	42	7	Male
	Missense	100	32	Female
	Missense	122	12	Female
	Missense	132	4	Male
	Missense	138	8	Female
	Missense	153	6	Female
	Missense	183	7	Female
	Missense	214	8	Male
	Missense	217	10	Male
	Missense	104	21	Male
	Missense	215	7	Male
	Missense	243	18	Male
	Missense	110	8	Male
	Missense	160	9	Male
	Missense	227	3	Female
	Missense	36	18	Female
	Missense	253	18	Female
	Missense	53	11	Male
	Missense	94	16	Male
	Missense	134	6	Female
	Missense	125	9	Male
	Missense	174	4	Female
	Missense	96	16	Male
	Missense	14	19	Male
	Missense	23	8	Male
	Missense	70	9	Female
	Missense	105	15	Female
	Missense	226	5	Male
	Missense	111	4	Female
	Missense	109	5	Male
	Missense	101	7	Female
	Missense	241	6	Male
	Missense	212	17	Female
	Missense	173	15	Male
	Missense	190	9	Female

	Missense	207	14	Female
	Missense	228	4	Male
	Missense	18	13	Female
Null	Frameshift	130	7	Female
	Frameshift	88	33	Female
	Nonsense	24	7	Female
	Frameshift	249	7	Male
	Frameshift	159	4	Female
	Nonsense	197	7	Male
	Frameshift	90	8	Male
	Nonsense	144	8	Female
	Splice	1	7	Female
	Nonsense	39	13	Male
	Nonsense	27	14	Female
	Frameshift	5	10	Female
	Frameshift	28	16	Male
	Nonsense	55	38	Male
	Frameshift	196	33	Female
	Frameshift	16	6	Male
	Frameshift	73	15	Male
	Frameshift	218	4	Male
	Frameshift	250	10	Male
	Nonsense	264	6	Female
	Nonsense	170	13	Male
	Nonsense	114	8	Female
	Nonsense	195	6	Female
	Nonsense	224	3	Male
	Nonsense	252	27	Male
	Frameshift	65	16	Female
	Frameshift	10	19	Male
	Frameshift	123	6	Male
	Nonsense	4	5	Male
	Nonsense	89	3	Female
	Nonsense	117	15	Male
	Nonsense	166	30	Female
	Nonsense	192	7	Male
	Nonsense	219	4	Female
	Nonsense	83	7	Female
	Nonsense	127	11	Female
	Nonsense	97	9	Female
	Frameshift	235	4	Male
	Frameshift	205	8	Male
	Frameshift	46	14	Female

Frameshift	119	7	Female
Nonsense	106	4	Male
Frameshift	220	11	Female
Nonsense	20	11	Male
Splice	35	14	Female
Splice	112	6	Male
Frameshift	98	11	Female
Nonsense	6	13	Male
Nonsense	108	17	Male
Nonsense	29	6	Male
Frameshift	48	6	Male
Frameshift	158	6	Female
Nonsense	59	19	Male
Nonsense	60	19	Male
Nonsense	69	10	Male
Nonsense	165	7	Female
Nonsense	38	9	Female
Frameshift	79	20	Male
Frameshift	103	14	Female
Frameshift	75	10	Female
Frameshift	208	6	Female
Frameshift	120	14	Male
Frameshift	81	11	Male
Frameshift	7	12	Male
Frameshift	150	14	Male
Frameshift	3	12	Male
Frameshift	26	5	Male
Nonsense	179	8	Female
Nonsense	221	11	Female
Exonic deletion	78	9	Female
Exonic deletion	92	7	Male
Exonic deletion	107	8	Female
Exonic deletion	140	16	Male
Multiexon deletion	2	10	Female
Multiexon deletion	8	7	Female
Multiexon deletion	22	19	Female
Multiexon deletion	25	9	Female
Multiexon deletion	64	29	Male
Multiexon deletion	80	3	Female
Multiexon deletion	82	8	Male
Multiexon deletion	102	7	Male
Multiexon deletion	115	6	Female
Multiexon deletion	126	18	Male

	Multiexon deletion	143	3	Male
	Multiexon deletion	146	4	Female
	Multiexon deletion	149	20	Male
	Splice	34	16	Female
	Splice	86	24	Male
	Splice	87	20.5	Male
	Splice	95	4	Female
	Splice	157	11	Male
	Splice	182	6	Female
	Splice	187	5	Male
Chromosomal	Duplication	188	6	Male
	Duplication	56	7	Male
	Duplication	62	6	Male
	Contiguous deletion	239	3	Male
	Contiguous deletion	11	4	Female
	Contiguous deletion	44	12	Female
	Contiguous deletion	141	10	Female
	Contiguous deletion	12	17	Male
	Contiguous deletion	93	7	Male
	Contiguous deletion	74	7	Female
	Contiguous deletion	85	7	Female
	Contiguous deletion	37	3	Female
	Contiguous deletion	17	13	Male
	Contiguous deletion	21	12	Female
	Contiguous deletion	156	7	Male
	Contiguous deletion	45	5	Male
	Contiguous deletion	223	3	Male
	Contiguous deletion	77	14	Male
	Contiguous deletion	206	4	Female
	Contiguous deletion	147	22	Female
	Contiguous deletion	30	3	Male
	Contiguous deletion	177	35	Female
	Contiguous deletion	184	5	Female
	Contiguous deletion	200	6	Female
	Contiguous deletion	72	9	Female
Other	Intronic	178	10	Male

Genomic alteration	Protein	Origin
c.185T>A	p.Val62Asp	de novo
c.257T>G	p.Leu86Arg	de novo
c.287T>G	p.Leu96Arg	de novo
c.392T>A	p.Val131Glu	de novo
c.760C>T	p.His254Tyr	de novo
c.1136A>C	p.Gln379Pro	de novo
c.1165C>T	p.Arg389Cys	de novo
c.1165C>T	p.Arg389Cys	de novo
c.1165C>T	p.Arg389Cys	de novo
c.1165C>T	p.Arg389Cys	de novo
c.1165C>T	p.Arg389Cys	de novo
c.1165C>T	p.Arg389Cys	de novo
c.1165C>T	p.Arg389Cys	de novo
c.1165C>T	p.Arg389Cys	Unknown
c.1165C>T	p.Arg389Cys	Unknown
c.1165C>T	p.Arg389Cys	de novo
c.1166G>T	p.Arg389Leu	de novo
c.1166G>A	p.Arg389His	de novo
c.1166G>A	p.Arg389His	de novo
c.1169C>T	p.Thr390Ile	de novo
c.1195C>T	p.Arg399Cys	de novo
c.1195C>T	p.Arg399Cys	de novo
c.1196G>C	p.Arg399Pro	Unknown
c.1196G>C	p.Arg399Pro	de novo
c.1196G>A	p.Arg399His	de novo
c.1196G>A	p.Arg399His	de novo
c.1196G>A	p.Arg399His	Unknown
c.1196G>T	p.Arg399Leu	de novo
c.1226A>G	p.Gln409Arg	de novo
c.1253T>G	p.Met418Arg	de novo
c.1286G>A	p.Arg429Gln	de novo
c.1286G>A	p.Arg429Gln	de novo
c.1286G>A	p.Arg429Gln	de novo
c.1286G>A	p.Arg429Gln	Unknown
c.1286G>A	p.Arg429Gln	de novo
c.1541A>G	p.Gln514Arg	de novo
c.1554T>G	p.Cys518Trp	de novo
c.1564C>T	p.Arg522Cys	de novo
c.1564C>T	p.Arg522Cys	de novo
c.1634T>C	p.Leu545Pro	Unknown
c.1946C>T	p.Ser649Leu	de novo
c.1946C>T	p.Ser649Leu	de novo

c.1946C>T	p.Ser649Leu	de novo
c.1946C>T	p.Ser649Leu	de novo
c.1964C>T	p.Pro655Leu	de novo
c.9_16del	p.Arg4Glufs*48	de novo
c.19dup	p.Ser7Lysfs*68	de novo
c.124G>T	p.Gly42*	de novo
c.138del	p.Arg46Serfs13	de novo
c.163del	p.Val55Trpfs*4	de novo
c.318T>G	p.Tyr106*	Unknown
c.334del	p.Ala112Profs*6	de novo
c.337C>T	p.Gln113*	Parental mosaic
c.346G>C	p.Gly116Arg	de novo
c.346G>T	p.Gly116*	de novo
c.390T>A	p.Tyr130*	de novo
c.400del	p.Ala134Hisfs*17	de novo
c.482del	p.Lys161Serfs*19	de novo
c.505C>T	p.Gln169*	de novo
c.581_584del	p.Glu194Alafs*7	de novo
c.583dup	p.Cys195Leufs*14	de novo
c.594_595del	p.Gln199Glufs*9	de novo
c.622dup	p.Ser208Lysfs*20	de novo
c.642_643dup	p.Ser215Cysfs*45	Unknown
c.652A>T	p.Lys218*	Unknown
c.688A>T	p.Lys230*	Unknown
c.715C>T	p.Arg239*	de novo
c.715C>T	p.Arg239*	Unknown
c.715C>T	p.Arg239*	de novo
c.805G>T	p.Glu269*	Unknown
c.808_809del	p.Gln270Valfs*33	de novo
c.816del	p.His273Thrfs*21	de novo
c.832del	p.His278Thrfs*16	Unknown
c.847C>T	p.Arg283*	de novo
c.847C>T	p.Arg283*	de novo
c.847C>T	p.Arg283*	de novo
c.847C>T	p.Arg283*	de novo
c.847C>T	p.Arg283*	Unknown
c.847C>T	p.Arg283*	de novo
c.868C>T	p.Gln290*	de novo
c.988C>T	P.Gln330*	de novo
c.997C>T	p.Gln333*	de novo
c.1016del	p.Phe339Serfs*2	Unknown
c.1105_1106insT	p.Arg369Metfs*3	Unknown
c.1131_1132del	p.Ser378Profs*18	Unknown

c.1132_1133insAC	p.Ser378Tyrfs*36	de novo
c.1135C>T	p.Gln379*	de novo
c.1148del	p.Ala383Glufs*30	de novo
c.1171C>T	p.Gln391*	de novo
c.1174G>C	p.Gly392Arg	Parental mosaic
c.1175G>A	p.Gly392Glu	de novo
c.1196del	p.Arg399Leufs*14	de novo
c.1255C>T	p.Gln419*	de novo
c.1285C>T	p.Arg429*	de novo
c.1285C>T	p.Arg429*	de novo
c.1311_1314 dup	p.Arg439Glyfs*38	Unknown
c.1329_1347dup	p.Ser450Glnfs*32	Unknown
c.1375C>T	p.Arg459*	de novo
c.1375C>T	p.Arg459*	de novo
c.1375C>T	p.Arg459*	Unknown
c.1477C>T	p.Gln493*	de novo
c.1495A>T	p.Lys499*	de novo
c.1515del	p.Phe505Leufs*41	de novo
c.1511_1512insCAAGCCT	p.Phe505Lysfs*10	de novo
c.1592dupA	p.Asn531Lysfs*21	Unknown
c.1624dup	p.Arg542Profs*10	Unknown
[c.1639del; 1642C>T]	p.Leu547Phefs*77	de novo
c.1657del	p.Asp553Metfs*71	Unknown
c.1728del	p.Glu577Serfs*47	Unknown
c.1825_1826dup	p.Asp609Glufs*16	Unknown
c.1945dup	p.Ser649Phefs*40	de novo
c.2028del	p.Glu678Serfs*18	de novo
c.2074G>T	p.Glu692*	Unknown
c.2074G>T	p.Glu692*	Unknown
Exon 4	Intragenic Deletion	Unknown
Exon 7	Intragenic Deletion	Unknown
Exon 5	Intragenic Deletion	de novo
Exon 9	Intragenic Deletion	de novo
Exons 1-8	Intragenic Deletion	Presumed germline
Exons 9-10	Intragenic Deletion	Unknown
Exons 1-11	Intragenic Deletion	Unknown
Exons 5-12	Intragenic Deletion	de novo
Exons 5-8	Intragenic Deletion	Unknown
Exons 4-8	Intragenic Deletion	Unknown
Exons 8-9	Intragenic Deletion	Unknown
Exons 7-8	Intragenic Deletion	de novo
Exons 7-8	Intragenic Deletion	de novo
Exons 1-12	Intragenic Deletion	de novo

Exons 5-11	Intragenic Deletion	Unknown
Exons 5-7	Intragenic Deletion	Unknown
Exons 5-11	Intragenic Deletion	Unknown
c.1174-2A>G	p.?	de novo
c.598-2A>G	p.?	de novo
c.598-2A>G	p.?	de novo
c.473+1delG	p.?	Unknown
c.474-2A>G	p.?	de novo
c.344_346+1dup	p.?	de novo
c.1174-1G>A	p.?	Unknown
chr2: 200,188,500-200,286,593	182kb duplication	de novo
chr2: 200,239,708-203,172,190	3Mb duplication	Presumed germline
chr2:200,239,961-203,172,079	3Mb duplication	Presumed germline
chr2:199,834,462-200,608,662	774kb deletion	Unknown
chr2:199,637,244-201,405,505	1.8Mb deletion	Unknown
chr2:198,588,111-200,411,579	1.8Mb deletion	Unknown
chr2:198,478,190-201,386,084	2.9Mb deletion	Unknown
chr2:199,632,071-203,218,563	3.6Mb deletion	Unknown
chr2:197,975,602-201,688,027	3.7Mb deletion	Unknown
chr2:198,971,305-203,082,242	4.1Mb deletion	Unknown
chr2:199,675,235-204,047,452	4.4Mb deletion	Unknown
chr2: 198,918,163-203,562,881	4.6Mb deletion	Unknown
chr2:198,264,747-202,954,071	4.7Mb deletion	Unknown
chr2:196,233,275-201,079,054	4.8Mb deletion	Unknown
chr2:194,765,436-200,436,597	5.6Mb deletion	Unknown
chr2: 195,763,510-202,021,245	7.0Mb deletion	Unknown
chr2:196,190,290-203,880,785	7.7Mb deletion	Unknown
chr2:200,125,357-208,282,806	8.1Mb deletion	Unknown
chr2:190,345,272-200,212,289	9.6Mb deletion	Unknown
chr2:191,750,202-202,297,376	10.5Mb deletion	Unknown
chr2:198,658,273-213,766,678	15.0Mb deletion	Unknown
chr2:187,058,538-204,602,930	17.5Mb deletion	Unknown
chr2:183,039,526-201,581,757	18.5Mb deletion	Unknown
chr2:189,322,667-208,515,614	19.2Mb deletion	Unknown
chr2:183,387,076-207,078,533	23.7Mb deletion	Unknown
c.1741-8A>G	p.?	de novo

Previously published	Domain	aptive/Cognit	Verbal	:xpressive
PMID: 29436146	ULD	3	0	1
PMID: 31021519	ULD	0	0	1
PMID: 29436146	ULD	3	0	0
PMID: 29436146	ULD	5	3	3
PMID: 31021519	N/A	5	3	5
PMID: 31021519	CUT1	5	3	2
PMID: 31021519	CUT1	5	3	3
PMID: 31021519	CUT1	5	3	2
PMID: 31021519	CUT1	5	3	1
PMID: 31021519	CUT1	3	3	2
PMID: 31021519	CUT1	5	3	1
PMID: 31021519	CUT1	5	3	4
PMID: 31021519	CUT1	5	3	2
PMID: 31021519	CUT1	3	0	1
PMID: 31021519	CUT1	5	3	1
PMID: 31021519	CUT1	5	3	4
PMID: 31021519	CUT1	5	3	2
PMID 31302918	CUT1	3	3	2
PMID 31302918	CUT1	5	3	3
PMID: 31021519	CUT1	5	3	4
PMID: 33661512	CUT1	5	0	1
PMID: 33661512	CUT1	3	3	3
PMID: 31021519	CUT1	5	3	2
PMID: 31021519	CUT1	5	0	1
PMID: 31021519	CUT1	0	0	0
PMID: 31021519	CUT1	5	3	2
PMID: 31021519	CUT1	3	3	3
PMID: 31021519	CUT1	5	3	5
Unpublished	CUT1	5	3	3
PMID: 31021519	CUT1	5	3	3
PMID: 31021519	CUT1	5	3	4
PMID: 31021519	CUT1	5	3	4
PMID: 31021519	CUT1	5	0	1
PMID: 31021519	CUT1	5	3	3
PMID: 31021519	CUT1	5	3	4
PMID: 31021519	CUT2	5	3	4
PMID: 31021519	CUT2	5	0	2
PMID: 31021519	CUT2	0	0	0
PMID: 31021519	CUT2	0	0	0
Unpublished	CUT2	5	3	2
Unpublished	HOX	3	0	0
Unpublished	HOX	3	0	0

Unpublished	HOX	5	0	1
Unpublished	HOX	5	3	3
PMID: 31021519	HOX	5	3	4
PMID: 31021519	N/A	0	0	0
PMID: 31021519	N/A	3	0	0
PMID: 31021519	N/A	3	0	2
Unpublished	N/A	3	0	1
PMID: 32446642	N/A	0	3	3
Unpublished	ULD	5	0	2
PMID: 31021519	ULD	3	0	2
PMID: 33661512	ULD	3	0	1
PMID: 31021519	ULD	3	0	2
PMID: 31021519	ULD	5	3	3
PMID: 31021519	ULD	5	3	2
PMID: 31021519	ULD	3	3	1
PMID: 31021519	N/A	5	3	1
PMID: 31021519	CUTL	5	3	4
Unpublished	CUTL	5	3	1
PMID: 31021519	CUTL	5	3	2
PMID: 31021519	CUTL	5	3	4
Unpublished	CUTL	3	3	2
Unpublished	CUTL	5	3	4
Unpublished	CUTL	3	3	1
PMID: 28333917	CUTL	5	3	4
PMID: 31021519	N/A	5	3	0
PMID: 31021519	N/A	5	3	5
PMID: 31021519	N/A	5	3	4
Unpublished	N/A	5	3	4
PMID: 31021519	N/A	5	0	1
PMID: 31021519	N/A	3	3	1
PMID: 31021519	N/A	3	3	3
PMID: 31021519	N/A	0	0	1
PMID: 31021519	N/A	3	3	3
PMID: 31021519	N/A	5	3	2
PMID: 31021519	N/A	5	3	4
PMID: 31021519	N/A	5	3	3
PMID: 31021519	N/A	3	3	4
PMID: 31021519	N/A	5	3	3
PMID: 31021519	N/A	0	3	1
PMID: 31021519	N/A	5	3	0
Unpublished	N/A	3	3	2
Unpublished	CUT1	5	3	3
PMID: 31021519	CUT1	5	3	3

PMID: 31021519	CUT1	3	3	1
PMID: 31021519	CUT1	5	3	2
Unpublished	CUT1	5	0	1
PMID: 31021519	CUT1	5	3	1
PMID: 31021519	CUT1	5	3	2
PMID: 31021519	CUT1	3	3	3
PMID: 31021519	CUT1	5	3	3
PMID: 31021519	CUT1	5	3	1
PMID: 31021519	CUT1	3	3	1
PMID: 31021519	CUT1	5	3	5
PMID: 31021519	N/A	5	3	3
PMID: 33661512	N/A	0	0	1
PMID: 31021519	N/A	5	3	2
PMID: 31021519	N/A	5	3	3
PMID: 31021519	N/A	3	0	0
Unpublished	CUT2	5	3	4
PMID: 31021519	CUT2	3	0	0
PMID: 31021519	CUT2	5	3	3
PMID: 31021519	CUT2	5	0	1
PMID: 31021519	CUT2	5	3	1
Unpublished	CUT2	3	3	3
PMID: 31021519	CUT2	5	3	5
PMID: 31021519	CUT2	5	3	4
PMID: 31021519	N/A	5	3	1
Unpublished	N/A	3	0	0
PMID: 31021519	HOX	3	3	4
PMID: 31021519	N/A	3	3	4
PMID: 28151491	N/A	5	3	4
PMID: 28151491	N/A	5	3	5
PMID: 31021519	N/A	5	0	3
PMID: 31021519	N/A	5	3	3
PMID: 31021519	N/A	5	0	2
PMID: 31021519	N/A	5	3	1
PMID: 31021519	N/A	3	0	1
PMID: 31021519	N/A	5	3	1
PMID: 31021519	N/A	5	3	4
PMID: 31021519	N/A	3	3	1
PMID: 31021519	N/A	5	3	2
PMID: 31021519	N/A	5	3	4
PMID: 31021519	N/A	5	3	3
PMID: 31021519	N/A	5	3	3
PMID: 31021519	N/A	5	3	5
PMID: 31021519	N/A	5	0	1

PMID: 32446642	N/A	5	3	3
Unpublished	N/A	0	3	0
PMID: 33661512	N/A	5	3	1
PMID: 31021519	N/A	3	3	1
PMID: 31021519	N/A	5	3	4
PMID: 31021519	N/A	5	0	0
PMID: 31021519	N/A	5	3	4
Unpublished	N/A	5	3	4
Unpublished	N/A	5	0	1
Unpublished	N/A	5	3	4
Unpublished	N/A	3	0	1
PMID: 29436146	N/A	5	0	2
PMID: 29436146	N/A	5	0	2
Unpublished	N/A	3	0	1
PMID: 29436146	N/A	3	3	3
PMID: 29436146	N/A	5	3	3
Unpublished	N/A	3	0	0
PMID: 29436146	N/A	5	3	1
Unpublished	N/A	3	3	3
PMID: 29436146	N/A	5	3	3
Unpublished	N/A	5	3	2
PMID: 29436146	N/A	5	3	3
PMID: 29436146	N/A	5	3	2
PMID: 29436146	N/A	5	0	1
Unpublished	N/A	5	0	2
PMID: 29436146	N/A	5	3	1
Unpublished	N/A	3	3	4
PMID: 29436146	N/A	5	3	3
Unpublished	N/A	3	3	4
Unpublished	N/A	5	0	2
PMID: 29436146	N/A	5	3	4
Unpublished	N/A	5	3	4
Unpublished	N/A	5	3	4
Unpublished	N/A	5	3	5
PMID: 29436146	N/A	3	3	5
Unpublished	N/A	0	0	0

Ambulation	Behavior	Sleep	Palate	Sialorrhea	Strabismus	Scoliosis
1	0	0	0	0	2	0
1	1	3	0	0	0	0
1	0	0	0	1	0	0
1	1	0	2	0	0	0
3	0	0	0	1	0	0
1	0	0	0	1	0	0
2	4	3	0	1	0	0
1	1	2	2	1	0	0
1	2	0	0	0	2	1
1	3	0	0	2	1	0
1	0	1	0	1	1	1
1	3	1	2	2	0	0
2	0	0	0	2	1	0
1	1	1	0	2	0	0
1	1	0	0	1	1	0
1	1	1	0	2	2	0
1	2	1	0	1	0	0
1	0	1	0	1	0	0
1	2	1	2	1	0	1
2	0	1	0	1	1	0
1	0	3	0	1	0	0
1	1	1	0	2	2	0
2	2	2	2	0	2	1
1	1	0	0	0	0	0
1	2	1	0	1	0	0
1	2	1	0	1	0	1
1	1	2	0	0	0	0
1	1	0	0	1	0	0
2	2	1	0	0	2	0
2	1	0	0	1	0	1
1	2	3	0	2	1	0
1	1	3	0	1	0	0
1	3	0	0	1	0	0
1	1	1	0	1	1	0
1	1	0	0	1	2	0
2	0	3	0	0	1	2
2	3	2	0	0	0	0
1	0	0	0	0	1	0
1	0	0	0	0	0	0
1	4	0	2	2	2	0
1	2	2	0	0	0	0
1	2	0	0	1	0	0

1	1	0	2	1	1	1
1	2	2	0	0	1	0
1	3	0	0	1	0	0
1	0	0	2	0	0	0
1	1	1	2	0	1	0
1	0	0	0	1	0	0
1	2	1	2	1	2	0
1	0	1	0	0	0	0
0	0	0	0	1	0	0
1	1	1	2	0	0	0
1	3	3	0	1	0	0
1	1	2	0	0	0	0
1	0	1	2	1	0	0
3	1	1	0	2	0	0
1	1	0	0	0	0	0
1	1	0	2	1	1	0
2	0	1	0	2	1	0
1	2	2	2	0	1	0
2	2	0	2	1	0	0
1	3	0	2	0	0	0
1	4	0	2	1	0	0
0	0	0	0	0	0	0
0	2	3	2	1	1	0
1	2	3	2	0	1	0
1	0	1	0	1	2	0
1	3	2	2	1	1	1
1	0	0	0	0	0	0
1	3	2	2	1	0	0
1	2	2	2	0	0	0
1	1	0	0	2	0	0
1	1	0	2	1	1	0
0	1	0	0	1	0	0
1	0	1	0	1	0	0
2	1	1	0	1	2	0
0	3	1	2	2	0	0
1	0	1	0	1	0	0
1	0	1	2	2	0	0
2	1	1	2	1	1	0
1	1	2	0	0	0	0
0	2	2	0	1	2	2
0	2	2	0	1	2	0
1	1	2	0	1	1	0
1	0	0	0	2	0	0

1	3	0	0	1	1	0
1	1	0	2	1	0	0
1	2	1	0	1	1	0
1	3	3	2	2	1	0
4	0	2	0	2	1	2
2	3	0	0	1	0	0
1	3	3	0	2	2	0
1	2	3	2	1	0	0
1	1	0	0	1	0	0
2	4	3	0	1	0	0
2	3	2	0	1	2	0
1	0	1	0	0	0	0
1	3	0	2	0	0	0
1	1	0	2	0	0	0
0	0	2	0	1	0	0
1	2	0	0	1	2	0
1	1	1	0	1	0	1
2	1	0	0	1	0	0
1	2	0	2	1	0	0
1	1	0	2	1	2	0
2	2	3	2	2	1	1
1	1	3	2	1	2	0
1	2	0	2	2	1	0
2	2	3	0	1	2	0
1	1	0	0	0	0	0
3	2	1	2	1	2	0
2	0	0	0	1	0	0
3	0	2	0	0	2	0
2	1	3	0	1	2	0
1	0	0	0	0	0	0
1	3	1	0	0	1	0
1	0	1	2	1	0	0
1	2	3	2	1	1	0
1	2	0	0	1	2	0
2	2	0	2	2	0	0
1	3	0	0	1	0	0
1	3	0	2	1	0	0
1	2	0	2	0	0	0
1	1	0	0	1	1	0
1	3	3	2	2	0	0
4	0	0	2	1	0	0
5	0	0	2	1	0	0
1	1	0	2	1	1	0

1	2	3	2	2	0	0
1	0	0	0	1	0	0
1	4	2	2	1	0	0
1	1	1	0	0	0	0
2	1	1	0	0	0	0
0	2	0	0	0	0	0
1	2	3	0	1	1	0
1	2	2	0	1	1	0
1	0	2	2	1	0	0
1	3	2	2	1	1	0
0	1	0	0	0	0	0
1	0	0	0	0	0	0
1	0	0	0	1	0	0
1	0	0	0	1	0	0
0	1	1	0	1	2	0
2	1	1	2	1	1	0
1	0	0	0	0	0	0
1	2	2	0	1	0	0
1	0	1	0	1	0	0
1	1	0	0	1	0	0
1	3	2	2	1	1	0
1	1	1	2	1	1	0
1	4	3	2	1	0	0
1	1	2	0	0	2	0
1	1	0	0	0	1	0
1	1	1	2	1	0	0
1	1	1	2	1	0	0
2	2	2	0	1	0	0
1	0	0	2	0	0	0
2	2	2	2	0	2	1
2	1	0	2	0	0	0
1	1	2	0	1	0	1
1	0	0	2	0	2	0
5	1	1	2	1	0	0
2	3	1	2	2	2	0
1	0	0	0	0	0	0

Muscle Tone	Bone Health	Growth/Feeding	Seizures	Dental
1	0	0	0	4
1	0	1	1	2
1	0	0	0	4
1	1	1	0	3
1	1	3	2	1
0	1	1	1	5
1	0	1	1	5
0	1	0	0	3
1	3	0	1	3
0	0	1	1	3
1	0	0	0	2
1	0	2	0	5
1	1	1	1	2
1	1	0	0	3
1	1	0	0	0
1	0	1	0	2
0	0	0	0	2
1	0	0	0	1
1	1	0	1	4
1	0	2	0	1
0	1	0	0	3
0	0	0	1	0
0	3	0	0	2
1	0	0	0	3
1	0	1	0	5
0	0	1	0	2
0	0	0	1	2
2	2	1	0	1
1	0	0	0	2
0	3	0	1	3
1	0	2	1	4
0	1	1	1	5
1	0	1	0	4
1	0	0	1	4
1	0	0	0	3
0	3	3	2	4
0	1	1	2	1
1	1	0	0	3
1	0	0	0	0
1	1	1	0	3
0	0	0	0	4
0	0	0	0	1

0	0	0	0	2
1	0	0	0	0
1	0	0	0	4
0	0	0	0	3
1	0	0	0	2
1	1	0	0	5
1	2	0	0	3
1	0	0	0	4
0	0	0	0	0
1	0	0	0	1
1	0	0	0	2
1	0	2	0	5
1	2	0	0	5
1	1	0	0	4
1	0	0	0	5
0	0	0	0	2
1	0	0	0	1
0	1	0	0	4
1	3	0	0	2
0	0	0	0	3
1	0	0	0	3
1	1	0	0	4
1	0	0	1	4
0	0	0	0	1
1	0	0	0	0
0	0	0	0	2
1	1	3	0	4
0	0	0	1	3
0	1	1	0	4
1	0	1	0	2
0	0	0	0	3
1	2	0	0	5
1	1	0	0	2
1	3	0	0	2
0	0	0	0	1
1	0	0	0	1
1	0	0	0	1
1	1	1	0	2
0	0	2	0	5
1	0	0	0	2
1	0	0	0	1
1	2	2	1	2
1	1	1	2	3

1	0	0	0	4
1	3	1	0	2
1	0	0	0	1
1	1	0	0	5
1	3	3	0	2
1	1	3	2	1
1	0	0	1	3
1	1	0	0	4
0	1	0	0	3
1	2	3	1	4
1	0	0	0	4
0	0	0	0	2
0	0	0	0	5
0	0	0	0	5
0	0	2	0	5
0	0	0	2	1
1	2	1	1	5
1	0	3	0	4
1	0	1	0	4
1	1	3	1	3
1	1	0	2	2
0	1	0	0	1
1	1	1	0	4
1	0	0	1	2
0	0	0	0	3
1	0	1	0	3
0	2	1	0	3
1	0	1	0	5
0	1	0	0	3
0	0	0	1	5
1	1	0	1	2
0	0	1	0	4
1	1	1	1	4
1	2	1	1	5
1	0	3	0	3
1	3	0	0	5
1	0	2	0	3
0	3	0	0	3
1	0	0	0	1
0	2	0	0	2
1	1	2	0	3
1	2	3	1	4
0	0	0	0	5

1	0	0	2	3
0	0	0	0	3
0	1	0	0	5
0	0	0	1	4
0	1	2	0	4
1	0	0	0	3
0	3	2	0	4
0	0	1	0	5
0	0	0	0	4
1	0	0	0	1
0	0	0	0	1
0	2	1	0	5
0	1	1	0	5
0	0	0	0	2
1	0	0	0	3
1	0	2	0	4
0	1	0	0	2
0	0	0	1	5
1	0	1	0	4
0	0	1	0	4
1	1	0	0	2
1	0	0	0	3
1	2	2	0	5
0	1	1	1	5
1	0	1	0	1
1	0	3	0	2
0	1	2	1	2
1	0	2	1	4
0	0	0	0	0
0	0	2	1	2
1	0	1	1	4
2	1	0	0	3
1	0	3	0	1
1	0	2	1	1
1	0	2	0	3
1	0	1	0	1

Neurodevelopmental Max	Clinical Max	Total Max
5	7	12
6	5	11
4	6	10
13	8	21
16	9	25
11	9	20
20	9	29
14	7	21
12	11	23
12	8	20
11	6	17
17	12	29
12	9	21
7	7	14
11	4	15
15	8	23
14	3	17
10	3	13
15	11	26
15	6	21
10	5	15
12	5	17
16	10	26
8	4	12
4	8	12
14	5	19
13	3	16
15	7	22
16	5	21
14	9	23
18	11	29
17	9	26
10	7	17
14	8	22
14	7	21
12	15	27
14	5	19
1	6	7
1	1	2
15	12	27
8	4	12
6	2	8

8	7	15
16	2	18
16	6	22
1	5	6
6	6	12
6	8	14
8	11	19
8	5	13
7	1	8
8	4	12
11	4	15
9	8	17
13	11	24
15	8	23
9	6	15
11	6	17
15	5	20
14	8	22
14	9	23
16	5	21
13	7	20
12	6	18
12	10	22
18	4	22
10	4	14
19	7	26
13	9	22
18	7	25
11	8	19
9	6	15
11	7	18
2	9	11
11	5	16
14	9	23
16	5	21
13	3	16
12	6	18
15	9	24
8	7	15
12	8	20
12	5	17
15	10	25
12	10	22

11	7	18
12	10	22
10	4	14
16	12	28
16	14	30
14	9	23
18	9	27
15	9	24
9	5	14
22	12	34
18	8	26
3	2	5
14	7	21
13	7	20
5	8	13
15	6	21
6	12	18
14	9	23
9	9	18
11	14	25
16	12	28
18	7	25
15	12	27
16	7	23
5	3	8
16	10	26
12	7	19
17	9	26
19	7	26
9	6	15
16	6	22
9	8	17
15	12	27
7	13	20
13	11	24
16	10	26
11	9	20
13	8	21
14	4	18
18	8	26
15	10	25
18	14	32
8	9	17

17	10	27
4	4	8
16	9	25
10	5	15
16	7	23
7	4	11
18	11	29
17	8	25
9	7	16
18	6	24
5	1	6
8	8	16
8	8	16
5	3	8
11	7	18
15	11	26
4	3	7
14	7	21
11	7	18
13	6	19
16	8	24
14	8	22
18	13	31
10	10	20
9	4	13
12	9	21
13	9	22
17	9	26
11	2	13
13	10	23
15	9	24
16	8	24
13	9	22
20	8	28
17	12	29
1	3	4
